# The connectivity indices concept of neutrosophic graph and their application of computer network, highway system and transport network flow

**DOI:** 10.1038/s41598-024-54104-x

**Published:** 2024-02-28

**Authors:** M. Kaviyarasu, Muhammad Aslam, Farkhanda Afzal, Maha Mohammed Saeed, Arif Mehmood, Saeed Gul

**Affiliations:** 1 Department of Mathematics, Vel Tech Rangarajan Dr. Sagunthala R & D Institute of Science and Technology, Chennai, Tamil Nadu 600 0062 India; 2 Department of Mathematics, College of Sciences, King Khalid University, 61413 Abha, Saudi Arabia; 3 Department of Humanities and Basic Sciences, MCS, National University of Sciences and Technology, Islamabad, Pakistan; 4 Department of Mathematics, Faculty of Sciences, King Abdulaziz University, P.O. Box 80203, 21589 Jeddah, Saudi Arabia; 5 Department of Mathematics, Institute of Numerical Sciences, Gomal University, Dera Ismail Khan, 29050 KPK Pakistan; 6 Faculty of Economics, Kardan University, Parwan-e-Du Square, Kabul, Afghanistan

**Keywords:** Engineering, Mathematics and computing

## Abstract

To address information ambiguities, this study suggests using neutrosophic sets as a tactical tool. Three membership functions (called $$T_r, I_n, $$ and $$ F_i$$) that indicate an object’s degree of truth, indeterminacy, and false membership constitute the neutrosophic set. It becomes clear that the neutrosophic connectivity index (*CIN*) is an essential tool for solving practical problems, especially those involving traffic network flow. To capture uncertainties, neutrosophic graphs are used to represent knowledge at different membership levels. Two types of $$CIN_s,$$ mean *CIN* and *CIN*, are investigated within the framework of neutrosophic graphs. In the context of neutrosophic diagrams, certain node types-such as neutrosophic neutral nodes, neutrosophic connectivity reducing nodes (*NCRN*) , and neutrosophic graph connectivity enhancing nodes (*NCEN*) , play important roles. We concentrate on two types of networks, specifically traffic network flow, to illustrate the real-world uses of *CIN*. By comparing results, one can see how junction removal affects network connectivity using metrics like Connectivity Indexes (*CIN*) and Average Connectivity Indexes (*ACIN*) . A few nodes in particular, designated by ACIN as Non-Critical Removal Nodes $$ (NCRN_s) $$, show promise for increases in average connectivity following removal. To fully comprehend traffic network dynamics and make the best decisions, it is crucial to take into account both *ACIN* and *CIN* insights. This is because different junctions have different effects on average and overall connectivity metrics.

## Introduction

Zadeh^1^ introduced the idea of a fuzzy set by assigning degrees of membership between 0 and 1 to the elements of the set. In 1975, Rosenfeld^2^ examined the fuzzy graph. Thereafter, the identical idea was separately offered over the same time period by Yeh and Bang^3^. While Yeh and Bang provided applications for the concept of a fuzzy connected graph and^4–11^, Rosenfeld identified a few basic features. Mathew and Sunitha introduced arc types^12^ to fuzzy terminal nodes^13^ and geodesics^14^. Atanassov^15^ introduced the intuitionistic fuzzy set in 1986, which is a development of the fuzzy set. The intuitionistic fuzzy graph described and elaborated by Parvathi and Karunambigai^16^ and^17–20^ is a generalisation of the fuzzy graph. Karunambigai and Buvaneswari^21^ introduced slurs in *IFG*,  like strong and weakest arcs, strong path, $\alpha ,\beta$-strong, and $\gamma$-weak arcs. Karunambigai and Kalaivani^22^ viewed *IFGs* as a matrix representation. The cyclic $CI_9{N}$ and the mean cyclic $CI_{N}$ of fuzzy sets are two connectivity measurements that Binu et al. introduced^23^. The ideas of $CI_{N},$ average $CI_{N},$ and connectivity node types were introduced by Poulik and Ghorai to the bipolar fuzzy graph environment with applications^24^. $CI_{N}$ and $ACI_{N}$ were presented by Mathew and Mordeson^25^, who also explored their characteristics and practical uses. With a focus on illegal immigration networks, Binu et al.^26^ examined the Wiener index idea and the connection between the Wiener index and the connectedness index. Abdu Gumaei et al.^27^ presented *IFG* connectivity indices and their applications. Many researches using CI in intuitionisitic fuzzy graphs (See^28–34^). Tulat Naeem et al.^35^ established the notion of wiener index of intuitionistic fuzzy graphs with an application to transport network flow and^36^. A generalisation of the fuzzy set and the intuitionistic fuzzy set, the neutrosophic set was proposed by Smarandache^9^. It has the ability to interpret information that is hazy, unclear, and inconsistent. The idea of single-valued neutrosophical set (*SVNS*), a subclass of the neutrosophical set in which each membership of truth, indeterminacy, and falsehood accepts values between [0, 1], was then put forward by Wang et al.^37^. Strong, complete, and regular monovalent bipolar neutrosophic graphs were characterised and new findings on monovalent bipolar neutrosophic graphs were presented by Broumi et al.^38^ while Hassan et al. established a number of distinct types of bipolar neutrosophic graphs^39–41^. Merkepci and Ahmad^42^ introduced the notion on the conditions of imperfect neutrosophic duplets and imperfect neutrosophic triplets. Chakraborty et al.^43,44^ presented the concepet of De-neutrosophication technique of pentagonal neutrosophic number and application in minimal spanning tree, then Cylindrical neutrosophic single-valued number and its application in networking problem, multi-criterion group decision-making problem and graph theory.The author discussed, n-Super Hyper Graph and Plithogenic n-Super Hyper Graph, in Nidus Idearum in^45^ and Extension of Hyper Graph to n-Super Hyper Graph and to Plithogenic n-Super Hyper Graph, and Extension of Hyper Algebra to n-ary (Classical-/Neutro-/Anti-) Hyper Algebra, Further discussion on the said work was done in^46^. Celik and Hatip^47^ presented the concepet on the refined AH-Isometry and its applications in refined neutrosophic surfaces galoitica^43^; then Celik and Olgun defined some basic properties of the classification of neutrosophic complex inner product spaces. Ghods and Rostami^48^ examined the wiener index and applications in the Neutrosophic graphs and compared this index with the connectivity index, which is one of the most important degree-based indicators. Mathematically, it seems neutrosophic logic is more generalized than intuitionistic fuzzy logic. AL-Omer et al.^49^ the complement of the highest resul to f multiplication of two neutrosophic graphs is determined and complement of the maximum product of a neutrosophic graph,the degree of a vertex is investigated.The complement of the maximum product of two normal neutrosophic graphs has several results that a represented and provided. Neutrosophic logic can be applied to any field, to provide the solution for indeterminacy problem. Many of the real-world data have a problem of inconsistency, indeterminacy, and incompleteness.

Fuzzy sets provide a solution for uncertainties, and intuitionistic fuzzy sets handle incomplete information, but both concepts have failed to handle indeterminate information. Neutrosophic sets provide a solution for both incomplete and indeterminate information. It has mainly three degrees of membership, namely, indeterminacy, and falsity.

**Table:** 

Related work with different component			
References	Year	Techniques used	Solved problem
^50^	2020	Intuitionistic fuzzy soft graphs	Gain and loss of vertices pair
^41^	2020	Neutrosophic trees	Connectivity index
^23^	2020	Fuzzy graphs	Cyclic connectivity index
^48^	2021	Neutrosophic graphs	Wiener index
^27^	2021	Intuitionistic fuzzy graphs	Connectivity indices
^51^	2022	Bipolar fuzzy incidence graph	Cyclic connectivity index
^52^	2022	Spherical fuzzy environment	Most effective COVID-19 virus protector by MCGDM technique
^53^	2023	Intuitionistic fuzzy graphs	Wiener index

In the all the above works *CI* was obtained for fuzzy and intuitionistic fuzzy graphs but in real time average connectivity index and also be interpreted in neutrosophic graph. As far, there exists no research work on the concept of Connectivity index in neutrosophic graphs until now. In order to fill this gap in the literature and motivated by papers^27,48,51^, we put forward a new idea concerning the Connectivity index of neutrosophic graphs.

### Motivation

The authors discovered that, to the best of their knowledge, no study has reported on the connectedness indices of neutrosophic graphs and their applications in transport network flow after becoming informed and motivated by the aforementioned works. The following explanations provide a rundown of this work’s main contributions:

1. It introduces the concepts of neutrosophic graphs. The notion of neutrosophic graphs is the focus of the first approach in the literature, which is made in this study.
2. It is investigated the significance of this new class of graphs and how to differentiate it from the other existing classes.
3. Additionally, the neutrosophic graph’s connection indices and average connectivity indices are established.

#### Need

The need for employing neutrosophic sets and connectivity indices are necessary because real-world problem solving is inherently complex and uncertain, especially when it comes to traffic network flow. Traditional methods frequently fail to capture the ambiguity and vagueness inherent in data. Using $$T_r, I_n,$$ and $$ F_i$$ functions, neutrosophic sets offer a complex and symmetric framework for representing uncertainty in a realistic way. This method is improved with the addition of neutrosophic connectivity indices, which enable a thorough representation of knowledge using neutrosophic graphs. Certain nodes, like *NCRN*, *NCEN*, and neutrosophic neutral, add granularity to the representation of network connectivity and affect traffic flow.Through the use of examples, the development of connectivity indices within the neutrosophic graph framework seeks to provide a more precise and flexible tool for resolving real-world traffic flow problems. The use of these techniques demonstrates the need for a practical strategy in the modeling and resolution of complex issues related to traffic network flow and other related fields.

### Novelty

1. To define neutrosophic graph.
2. To provide a new definition of neutrosophic connectivity index.
3. To define a neutrosophic connectivity index with edge and vertex.
4. comparing the numerical results for the average connection index with the neutrosophic connectivity index.

This may help us to make better decision. The investigation of neutrosophic graphs and a few related ideas is the focus of this study. Preliminary specifications for this job are outlined in Section “Preliminaries”. The $CI_{N}$ ideas and $CI_{N}$ bounds for neutrosophic sets are developed in Section “Neutrosophic connectivity index graph”. The $CI_{N}$ of neutrosophic sub graphs with deleted vertices and edges is shown in Sections “Connectivity index with edge and vertex deleted neutrosophic graphs” and “Neutrosophic average connectivity index graph” discusses the $ACI_{N}$ and its attributes. In Section “Application of neutrosophic graph with transport network flow”, applications of $CI_{N}s$ are covered. real time applications for Section “Real life application”, in Section “Sensitivity analysis”, in Section “Comparative results”, in Section “Discussion of results”, Finally we discussed Advantages and Conclusion in Sections “Advantages and limitations” and “Conclusion” .

## Preliminaries

This section presents definitions and examples relating to neutrosophic graphs, arcs, in neutrosophic and neutrosophic cycles pertinent to the current work.

### Definition 1

^40^ A pair $$ G =(N,M)$$ is called a neutrosophic graph if,

1. $\check{\check{V}} = \lbrace u_{p_{1}}, u_{p_{2}},u_{p_{3}},\ldots ,u_{p_{n}} \rbrace$ with ${\check{\check{V}}} {\overset{T_{r}^{N}}{{\longrightarrow }} [0,1]},{\check{\check{V}}} {\overset{I_{n}^{N}}{{\longrightarrow }}[0,1]}$ and ${\check{\check{V}}} {\overset{F_{i}^{N}}{{\longrightarrow }}[0,1]}$ representing the truth-membership function, indeterminacy membership function and falsity membership function, $0 \le T_{r}^{N}(u_{p_{i}})+ I_{n}^{N}(u_{p_{i}})+ F_{i}^{N}(u_{p_{i}})\le 3$ for each $u_{p_{i}}\in \check{\check{V}}.$
2. $\check{\check{E}} \subseteq \check{\check{V}} \times \check{\check{V}}$ with $\check{\check{E}} \overset{T_{r}^{M}}{{\longrightarrow }} [0,1], \check{\check{E}} \overset{I_{n}^{M}}{{\longrightarrow }} [0,1],$ and $\check{\check{E}} \overset{F_{i}^{M}}{{\longrightarrow }} [0,1]$ being as follows:

$$\begin{aligned}{} & {} T_{r}^{M}({p_{i},u_{p_{j}}}) \le min \lbrace T_{r}^{N}(u_{p_{i}}),T_{r}^{N}(u_{p_{j}}) \rbrace \\{} & {} I_{n}^{M}({u_{p_{i}},u_{p_{j}}}) \le min \lbrace I_{n}^{N}(u_{p_{i}}),I_{n}^{N}(u_{p_{j}}) \rbrace \\{} & {} F_{i}^{M}({u_{p_{i}},u_{p_{j}}}) \ge max \lbrace F_{i}^{N}(u_{p_{i}}),F_{i}^{N}(u_{p_{j}}) \rbrace \end{aligned}$$

and $0 \le T_{r}^{M}(u_{p_{i}},u_{p_{j}})+I_{n}^{M}(u_{p_{i}},u_{p_{j}})+F_{i}^{M}(u_{p_{i}},u_{p_{j}})\le 3$ for all edge $(u_{p_{i}},u_{p_{j}}) \in E.$

### Definition 2

^40^ A neutrosophic graph *G* is complete if

$$\begin{aligned}{} & {} T_{r}^{M}({u_{p_{i}},u_{p_{j}}}) = min \lbrace T_{r}^{N}(u_{p_{i}}),T_{r}^{N}(u_{p_{j}})\rbrace \\{} & {} I_{n}^{M}({u_{p_{i}},u_{p_{j}}}) = min \lbrace I_{n}^{N}(u_{p_{i}}),I_{n}^{N}(u_{p_{j}})\rbrace \\{} & {} F_{i}^{M}({u_{p_{i}},u_{p_{j}}}) = max \lbrace F_{i}^{N}(u_{p_{i}}),F_{i}^{N}(u_{p_{j}})\rbrace \end{aligned}$$

for each $$ (u_{p_{i}},u_{p_{j}}) \in E.$$

Path has a significant and well-known part in neutrosophic graphs. We may define the path idea in neutrosophic graphs using the following definition.

### Definition 3

^38^ A neutrosophic graph with different vertices $u_{P_{1}}, u_{P_{2}},u_{P_{3}},\ldots ,u_{P_{n}}$ said to have a path $\check{\check{V}}$,if it met one of the conditions below.

1. $$ T_{r}^{M}(u_{p_{i}},u_{p_{j}})>0,\, I_{n}^{M}(u_{p_{i}},u_{p_{j}})>0,\, F_{i}^{M}(u_{p_{i}},u_{p_{j}})=0 $$
2. $$ T_{r}^{M}(u_{p_{i}},u_{p_{j}}) = 0,\, I_{n}^{M}(u_{p_{i}},u_{p_{j}}) = 0,\, F_{i}^{M}(u_{p_{i}},u_{p_{j}})>0 $$
3. $$ T_{r}^{M}(u_{p_{i}},u_{p_{j}})>0,\, I_{n}^{M}(u_{p_{i}},u_{p_{j}})>0,\, F_{i}^{M}(u_{p_{i}},u_{p_{j}})<0.$$

The neutrosophic graphical representations play a significant role to analyse the strength of the paths of vertices in a two dimensional space. The limitations of the strength of the paths have been defined component wise as well as total strength wise in the following statements discussed in the Definition 4:

### Definition 4

^38^ Assume taht a neutrosophic graph *G* contain a path $\check{\check{V}} = u_{p_{1}}, u_{p_{2}},u_{p_{2}},\ldots ,u_{p_{n}}$. Then $\check{\check{P}}$ is defined by

1. $$ T_{r} $$-strenthgh if $$ S_{T_{r}}= min \lbrace T_{r}^{M}(u_{p_{i}},u_{p_{j}}) \rbrace $$
2. $$ I_{n} $$-strenthgh if $$ S_{I_{n}}= min \lbrace I_{n}^{M}(u_{p_{i}},u_{p_{j}})\rbrace $$
3. $$ F_{i} $$-strenthgh if $$ S_{F_{i}}= max \lbrace F_{i}^{M}(u_{p_{i}},u_{p_{j}}) \rbrace $$
4. The $$ S_{\check{\check{P}}}=(S_{T_{r}},S_{I_{n}},S_{F_{i}}) $$ is said to be a $$ \check{\check{P}} $$ strength if both $$ S_{T_{r}}, S_{I_{n}}$$ and $$ S_{F_{i}} $$ to the same edge occur.

### Definition 5

^39^ The $T_{r}$-strength of connecting vertices $u_{p_{i}}$ and $u_{p_{j}}$ is define by $CONN_{T_{r}({\c{G}})}(u_{p_{i}},u_{p_{j}})= max \lbrace S_{T_{r}} \rbrace ,$ $I_{n}$-strength of connecting vertices $u_{p_{i}}$ and $u_{p_{j}}$ is define by $CONN_{I_{n}^{N}({\c{G}})}(u_{p_{i}},u_{p_{j}})= max \lbrace S_{I_{n}}\rbrace$ and $F_{i}$-strength of connecting vertices $u_{p_{i}}$ and $u_{p_{j}}$ is define by $CONN_{F_{i}({\c{G}})}(u_{p_{i}},u_{p_{j}})= min \lbrace S_{F_{i}} \rbrace$ for all possible paths between $u_{p_{i}}$ and $u_{p_{j}}$, where $CONN_{T_{r}({\c{G}})-(u_{p_{i}},u_{p_{j}})}(u_{p_{i}},u_{p_{j}}), CONN_{I_{n}({\c{G}})-(u_{p_{i}},u_{p_{j}})}(u_{p_{i}},u_{p_{j}})$ and $CONN_{F_{i}({\c{G}})-(u_{p_{i}},u_{p_{j}})}(u_{p_{i}},u_{p_{j}})$ denotes the $T_{r}, I_{n}$ and $F_{i}$-strength of connected with $u_{p_{i}}$ and $u_{p_{j}}$ achieved by eliminating the $(u_{p_{i}},u_{p_{j}})$ edge from ${\c{G}}$.

### Definition 6

^40^ An edge $(u_{p_{i}},u_{p_{j}})$ in a neutrosophic graph

1. strongest, if $T_{r}^{M}(u_{p_{i}},u_{p_{j}})\ge CONN_{T_{r}({\c{G}})}(u_{p_{i}},u_{p_{j}}), I_{n}^{M}(u_{p_{i}},u_{p_{j}})\ge CONN_{I_{n}({\c{G}})}(u_{p_{i}},u_{p_{j}})$ and $F_{i}^{M}(u_{p_{i}},u_{p_{j}})\le CONN_{F_{i}({\c{G}})}(u_{p_{i}},u_{p_{j}})$ for each $u_{p_{i}},u_{p_{j}}\in V.$
2. Weakest, if $T_{r}^{M}(u_{p_{i}},u_{p_{j}})< CONN_{T_{r}({\c{G}})}(u_{p_{i}},u_{p_{j}}), I_{n}^{M}(u_{p_{i}},u_{p_{j}})< CONN_{I_{n}({\c{G}})}(u_{p_{i}},u_{p_{j}})$ and $F_{i}^{M}(u_{p_{i}},u_{p_{j}})>CONN_{F_{i}({\c{G}})}(u_{p_{i}},u_{p_{j}})$ for each $u_{p_{i}},u_{p_{j}}\in V.$

### Definition 7

^41^ Let {\c{G}}=(Ņ,M̧) be a neutrosophic graph. A path $$ P:u_{p_{i}}-u_{p_{j}}$$ in {\c{G}}is said to be a strong path if *P* consists of only strong edges.

**Figure 1:**
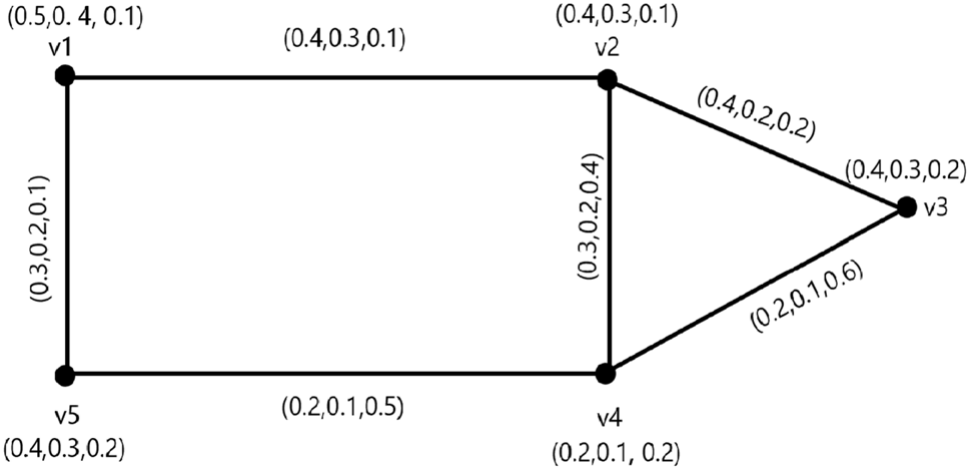
Neutrosophic graph with strong and weakest arcs.

### Example 1

In Fig. 1, $T_{r}^{M}(v_{p_{1}},v_{p_{2}})= 0.7 = CONN_{T_{r}({\c{G}})}(v_{p_{1}},v_{p_{2}}), I_{n}^{M}(v_{p_{1}},v_{p_{2}})= 0.6 = CONN_{I_{n}({\c{G}})}(v_{p_{1}},v_{p_{2}})$ and $F_{i}^{M}(v_{p_{1}},v_{p_{2}})= 0.3 = CONN_{F_{i}({\c{G}})}(v_{p_{1}},v_{p_{2}})$, which is implies that $(v_{p_{1}},v_{p_{2}})$ is a strong arc. Similarly,$(v_{p_{2}},v_{p_{3}}),(v_{p_{1}},v_{p_{5}}),(v_{p_{2}},v_{p_{4}}),$ are strong arc and $(v_{p_{3}},v_{p_{4}}),(v_{p_{4}},v_{p_{5}})$ are weakest arcs.

In this regard, $P = v_{p_{1}}v_{p_{2}}v_{p_{3}}$ is a strong path.

### Definition 8

^53^ An arc $(u_{p_{i}},u_{p_{j}})$ in a neutrosophic graph ({\c{G}})=(Ņ,M̧) is

1. If $$ T_{r}^{M}(u_{p_{i}},u_{p_{j}})>CONN_{T_{r}({\c{G}})-(u_{p_{i}},u_{p_{j}})}(u_{p_{i}},u_{p_{j}}),$$
  $I_{n}^{M}(u_{p_{i}},u_{p_{j}})>CONN_{I_{n}({\c{G}})-(u_{p_{i}},u_{p_{j}})}(u_{p_{i}},u_{p_{j}})$ and $F_{i}^{M}(u_{p_{i}},u_{p_{j}})<CONN_{F_{i}({\c{G}})-(u_{p_{i}},u_{p_{j}})}(u_{p_{i}},u_{p_{j}})$ is called $\alpha$-strong.
2. If $$ T_{r}^{M}(u_{p_{i}},u_{p_{j}})= CONN_{T_{r}({\c{G}})-(u_{p_{i}},u_{p_{j}})}(u_{p_{i}},u_{p_{j}}),$$
  $$I_{n}^{M}(u_{p_{i}},u_{p_{j}})= CONN_{I_{n}({\c{G}})-(u_{p_{i}},u_{p_{j}})}(u_{p_{i}},u_{p_{j}}) $$
  and $F_{i}^{M}(u_{p_{i}},u_{p_{j}})= CONN_{F_{i}({\c{G}})-(u_{p_{i}},u_{p_{j}})}(u_{p_{i}},u_{p_{j}})$ is called $\beta$-strong.
3. If $$ T_{r}^{M}(u_{p_{i}},u_{p_{j}})< CONN_{T_{r}({\c{G}})-(u_{p_{i}},u_{p_{j}})}(u_{p_{i}},u_{p_{j}}),$$
  $$I_{n}^{M}(u_{p_{i}},u_{p_{j}})< CONN_{I_{n}({\c{G}})-(u_{p_{i}},u_{p_{j}})}(u_{p_{i}},u_{p_{j}}) $$
  and $F_{i}^{M}(u_{p_{i}},u_{p_{j}})> CONN_{F_{i}({\c{G}})-(u_{p_{i}},u_{p_{j}})}(u_{p_{i}},u_{p_{j}})$ is called $\gamma$-weak.

**Figure 2:**
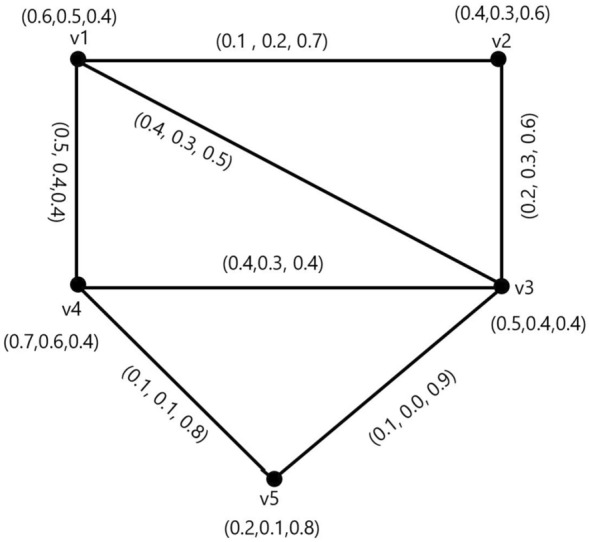
A Neutrosophic graph with $\alpha ,\beta$-strong, and $\gamma$-weakest arcs.

### Example 2

Figure 2, shows, the arcs $(v_{p_{1}},v_{p_{4}}),(v_{p_{2}},v_{p_{3}}),(v_{p_{4}},v_{p_{5}}),(v_{p_{1}},v_{p_{3}})$ are $\alpha$-strong, $(v_{p_{3}},v_{p_{4}})$ is $\beta$-strong, and $(v_{p_{1}},v_{p_{2}}),(v_{p_{3}},v_{p_{5}})$ are $\gamma$-weak.

### Definition 9

^41^ A path in a neutrosophic graph containing only $\alpha  \& \beta$-strong arc are called $\alpha  \&  \beta$-strong.

### Definition 10

^41^

1. If ${\c{G}}^{*}=(N^{*},M{*})$ is a cycle, then {\c{G}}=(Ņ,M̧) is said to be a cycle
2. If ${\c{G}}^*=(N^{*},M{*})$ is cycle, and $\not \exists$ a pair $(x,y)\in M^{*}$ be such that $T_{r}^{M}(t,x)= min \lbrace T_{r}^{M}(a,b) \mid (a,b)\in M^{*}\rbrace , I_{n}^{M}(t,x)= min \lbrace I_{n}^{M}(a,b)\mid (a,b)\in M^{*}\rbrace ,$ and $F_{i}^{M}(t,x)= max \lbrace F_{i}^{M}(a,b)\mid (a,b)\in M^{*}\rbrace ,$ then ${\c{G}}$ is said to be a neutrosophic cycle.

### Example 3

**Figure 3:**
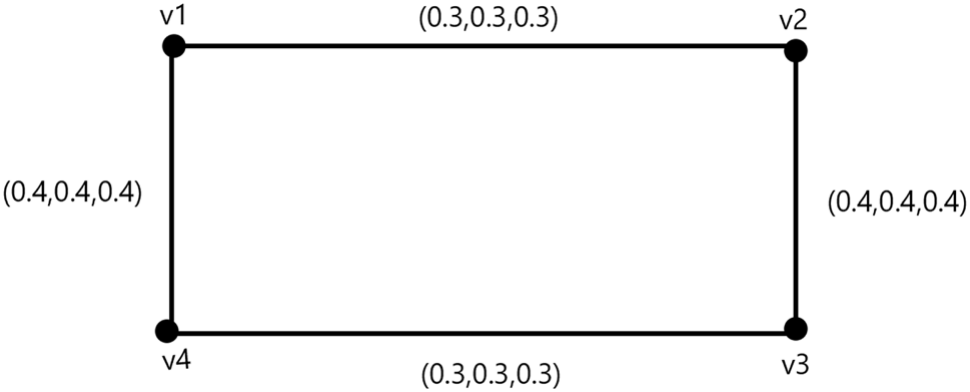
A neutrosophic graph cycle.

In Fig. 3 we consider $T_{r}^{N}(u_{p}),I_{n}^{N}(u_{p}),F_{i}^{N}(u_{p})= (.3,.3,.4), \forall u\in N^{*}.$Then, $min \lbrace T_{r}^{M}(u_{p},v_{p})\rbrace =0.3, min\lbrace I_{n}^{M}(u_{p},v_{p})\rbrace =0.3$ and $max \lbrace F_{i}^{M}(u_{p},v_{p})\rbrace =0.4$.

Our study’s primary goal is to increase the precision and accuracy of topological indices research, specifically in the perspective of connection indices. Neutrosophic graphs provide more information than intuitionistic fuzzy graphs. In a certain condition of haziness and ambiguity, intuitionistic are termed by having membership grade and non-membership grade, but neutrosophic graphs are considered by the 3 grades, namely true, indeterminacy, and false membership grades. As we are using three membership grades, neutrosophic graphs do not have as much loss of information as relating to intutionistic graphs. For this reason, we would want to suggest different CIN models for neutrosophic graphs and learn how to use them.

## Neutrosophic connectivity index graph

Naturally, when we discuss a network similar to a transport network, we consider its connectedness. The network’s connectedness indicates how stable and dynamic it is. Therefore, we may claim that this connectedness metric is the most fundamental and essential. The connection metric is already present in neutrosophic graphs. However, neutrosophic graph is an extension of intuitionistic fuzzy graph, performs better when intuitionistic fuzzy graphs are not permeable. The authors have thus suggested this notion of connectedness from intuitionistic fuzzy graphs to neutrosophic graphs for the above mentioned purpose.The authors have given some findings about the connectivity of neutrosophic graphs.

### Definition 11

The $CI_{N}$ of a neutrosophic graph {\c{G}}=(Ņ,M̧) is demarcated as

1 $$\begin{aligned} CI_{N}({\c{G}})= & {} \sum _{u_{p},v _{p}\in \check{\check{V}}({\c{G}})}(T_{r}^{N}(u_{p}),I_{n}^{N}(u_{p}),F_{i}^{N}(u_{p}))(T_{r}^{N}(v_{p}),I_{n}^{N}(v_{p}),F_{i}^{N}(v_{p})) \times CONN_{{\c{G}}}(u_{p},v_{p}) \nonumber \\= & {} \sum _{u_{p},v _{p}\in \check{\check{V}}({\c{G}})}(T_{r}^{N}(u_{p}),I_{n}^{N}(u_{p}),F_{i}^{N}(u_{p}))(T_{r}^{N}(v_{p}),I_{n}^{N}(v_{p}),F_{i}^{N}(v_{p}))\nonumber \\{} & {} \>(CONN_{T_{r}({\c{G}})}(u_{p},v_{p}),CONN_{I_{n}({\c{G}})}(u_{p},v_{p}),CONN_{F_{i}({\c{G}})}(u_{p},v_{p})) \nonumber \\= & {} \sum _{u_{p},v _{p}\in \check{\check{V}}({\c{G}})}(T_{r}^{N}(u_{p})T_{r}^{N}(v_{p})CONN_{T_{r}({\c{G}})}(u_{p},v_{p}))\nonumber \\{} & {} +\>(I_{n}^{N}(u_{p})I_{n}^{N}(v_{p})CONN_{I_{n}({\c{G}})}(u_{p},v_{p})) +(F_{i}^{N}(u_{p})F_{i}^{N}(v_{p})CONN_{F_{i}({\c{G}})}(u_{p},v_{p})) \nonumber \\= & {} \sum _{u_{p},v _{p}\in \check{\check{V}}({\c{G}})} T_{r}^{N}(u_{p})T_{r}^{N}(v_{p})CONN_{T_{r}({\c{G}})}(u_{p},v_{p}) \nonumber \\{} & {} +\>\sum _{u_{p},v _{p}\in \check{\check{V}}({\c{G}})}I_{n}^{N}(u_{p})I_{n}^{N}(v_{p})CONN_{I_{n}({\c{G}})}(u_{p},v_{p})\nonumber \\{} & {} +\>\sum _{u_{p},v _{p}\in \check{\check{V}}({\c{G}})}F_{i}^{N}(u_{p})F_{i}^{N}(v_{p})CONN_{F_{i}({\c{G}})}(u_{p},v_{p}) \end{aligned}$$

2 $$\begin{aligned}= & {} T_{r}CI_{N}({\c{G}})+I_{n}CI_{N}({\c{G}})+F_{i}CI_{N}({\c{G}}) \end{aligned}$$

where $T_{r}CI_{N}({\c{G}}),I_{n}CI_{N}({\c{G}})  \&  F_{i}CI_{N}({\c{G}})$ are $T_{r}, I_{n} \&  F_{i}$-connectivity index of ${\c{G}}$, and $CONN_{T_{r}({\c{G}})}(u_{p},v_{p}), CONN_{I_{n}({\c{G}})}(u_{p},v_{p})  \&  CONN_{F_{i}({\c{G}})}(u_{p},v_{p})$ are $T_{r}, I_{n}  \&  F_{i}$-strength $u_{p} -v_{p}$.

### Example 4

In Fig. 1,

$$\begin{aligned} T_{r}CI_{N}= & {} \sum _{u_{p},v _{p}\in \check{\check{V}}({\c{G}})}T_{r}^{N}(u_{p})T_{r}^{N}(v_{p})CONN_{T_{r}({\c{G}})}(u_{p},v_{p})\\= & {} (.5)(.4)(.2)+(.5)(.4)(.4)+(.5)(.2)(.3)+(.5)(.4)(.3)+(.4)(.4)(.4)\\{} & {} \hspace{1cm}+(.4)(.2)(.3)+(.4)(.4)(.3)+(.4)(.2)(.3)+(.4)(.4)(.3)+(.2)(.4)(.3)\\= & {} 0.442 \\ I_{n}CI_{N}= & {} \sum _{u_{p},v _{p}\in \check{\check{V}}({\c{G}})} I_{n}^{N}(u_{p})I_{n}^{N}(v_{p})CONN_{I_{n}({\c{G}})}(u_{p},v_{p})\\= & {} (.4)(.3)(.1)+(.4)(.3)(.2)+(.4)(.1)(.2)+(.4)(.3)(.2)+(.3)(.2)(.2)\\{} & {} \hspace{1cm}+(.3)(.1)(.2)+(.3)(.3)(.2)+(.3)(.1)(.2)+(.3)(.3)(.2)+(.1)(.3)(.2)\\= & {} 0.140 \\ F_{i}CI_{N}= & {} \sum _{u_{p},v _{p}\in \check{\check{V}}({\c{G}})} F_{i}^{N}(u_{p})F_{i}^{N}(v_{p})CONN_{F_{i}({\c{G}})}(u_{p},v_{p})\\= & {} (.1)(.1)(.5)+(.1)(.2)(.2)+(.1)(.2)(.4)+(.1)(.2)(.1)+(.1)(.2)(.2)\\*[-2mm]{} & {} \hspace{1cm}+(.1)(.2)(.4)+(.1)(.2)(.1)+(.2)(.2)(.4)+(.2)(.2)(.2)+(.2)(.2)(.4)\\= & {} 0.073\\ CI_{N}= & {} T_{r}CI_{N}+I_{n}CI_{N}+F_{i}CI_{N} \\ CI_{N}= & {} 0.442+0.140+0.073\\ CI_{N}= & {} 0.635 \end{aligned}$$

It may be observe that $T_{r}CI_{N}({\c{G}})> I_{n}CI_{N}({\c{G}}) > F_{i}CI_{N}({\c{G}})$,which show that the level of $F_{i}CI_{N}({\c{G}})$ is lower than the level of $I_{n}CI_{N}({\c{G}})$ is lower than the level of $T_{r}CI_{N}({\c{G}})$.

### Proposition 1

If {\c{G}}=(Ņ,M̧) is a complete neutrosophic graph with $$ N^{*}=\lbrace v_{p_{1}},v_{p_{2}},\ldots ,v_{P_{\kappa }} \rbrace $$ be such that $$  t_{1} \le t_{2} \le \cdots \le t_{n}, r_{1} \le r_{2} \le \cdots \le r_{\kappa }  \&  s_{1} \ge s_{2} \ge \cdots \ge s_{\kappa },$$ where $$ t_{p_{i}}= T_{r}^{N}(v_{p{i}}),$$ $$ r_{p_{i}}= I_{n}^{N}(v_{p{i}})$$, and $$ s_{_{i}}= F_{i}^{N}(v_{p{i}})$$,

3 $$\begin{aligned} CI_{N}({\c{G}})= & {} \sum _{p_{i}=1}^{\kappa -1} t_{i}^{2} \sum _{j=i+1}^{\kappa } t_{j}+\sum _{i=1}^{n-1} r_{i}^{2} \sum _{j=i+1}^{n} r_{j}+\sum _{i=1}^{\kappa -1} s_{i}^{2} \sum _{j=i+1}^{\kappa } s_{j}. \end{aligned}$$

### Proof

Assume that $v_{p_{1}}$ is the vertex with the lowest truth-membership value $t_{1}$. A complete neutrosophic graphis $CONN_{T_{r}({\c{G}})}(u_{p},v_{p}) = T_{r}^{M}(u_{p},v_{p}) \forall u_{p},v_{p} \in N^{*}$, so, $T_{r}^{M}(v_{p_{1}},v_{p{i}})=t_{1}; 2 \le v_{pi} \le \kappa$ and hence, $T_{r}^{N}(v_{p_{1}})T_{r}^{N}(v_{p{i}}) CONN_{T_{r}({\c{G}})}(v_{p_{1}},v_{p{i}})= t_{1}.t_{P_{i}}.t_{1} = t_{1}^{2}t_{P_{i}}; 2 \le p_{i} \le \kappa .$ we have

4 $$\begin{aligned} \sum _{p_{i}=2}^{\kappa }T_{r}^{N}(v_{p_{1}})T_{r}^{N}(v_{p_{i}})CONN_{T_{r}({\c{G}})}(v_{p_{1}},v_{p_{i}})= & {} \sum _{p_{i}=2}^{\kappa }t_{1}^{2} t_{p_{i}}, \end{aligned}$$

for $$ v_{p_{2}},$$ is

5 $$\begin{aligned} \sum _{p_{i}=3}^{\kappa }T_{r}^{N}(v_{p_{2}})T_{r}^{N}(v_{p_{i}})CONN_{T_{r}({\c{G}})}(v_{p_{2}},v_{p_{i}})= & {} \sum _{p_{i}=3}^{\kappa }t_{2}^{2} t_{p_{i}}, \end{aligned}$$

for $$ v_{p_{3}} $$ is

6 $$\begin{aligned} \sum _{p_{i}=4}^{\kappa }T_{r}^{N}(v_{p_{3}})T_{r}^{N}(v_{p{i}})CONN_{T_{r}({\c{G}})}(v_{p_{3}},v_{p{i}})= & {} \sum _{i=4}^{\kappa }t_{3}^{2} t_{i}, \end{aligned}$$

and for $v_{p_{\kappa -1}}$ is

7 $$\begin{aligned} \sum _{p_{i}= \kappa }^{\kappa }T_{r}^{N}(v_{p_{\kappa -1}})T_{r}^{N}(v_{p_{i}})CONN_{T_{r}({\c{G}})}(v_{\kappa -1},v_{p_{i}})= & {} \sum _{p_{i}= \kappa }^{\kappa } t_{\kappa -1}^{2} t_{p_{i}}. \end{aligned}$$

The result of combining the equations above is

8 $$\begin{aligned} T_{r}CI_{\kappa }^{N}({\c{G}})= & {} \sum _{p_{i}=2}^{\kappa } t_{1}^{2} t_{p_{i}}+ \sum _{p_{i}=3}^{\kappa } t_{2}^{2} t_{p_{i}}+\sum _{p_{i}=4}^{\kappa } t_{3}^{2} t_{p_{i}}+\cdots + \sum _{p_{i}= \kappa }^{\kappa } t_{\kappa -1}^{2} t_{p_{i}} \end{aligned}$$

9 $$\begin{aligned}= & {} \sum _{p_{i}=1}^{\kappa -1} t_{p_{i}}^{2} \sum _{p_{j}=p_{i}+1}^{\kappa } t_{p_{j}}. \end{aligned}$$

Suppose $$v_{p_{1}} $$ is the vertex with least indermatiance-membership value $$r_{1}$$. Then, for a complete neutrosophic graph, $$ CONN_{I_{n}({\c{G}})}(u_{p},v_{p}) = I_{n}^{M}(u_{p},v_{p})\forall u_{p},v_{p} \in N^{*} $$, So, $$I_{n}^{M}(v_{p_{1}},v_{p{i}})=r_{1}; 2 \le i \le n $$ and hence,$$I_{n}^{N}(v_{p_{1}})I_{n}^{N}(v_{p{i}}) CONN_{I_{n}^{N}({\c{G}})}(v_{p_{1}},v_{p{i}})= r_{1}.r_{i}.r_{1} = r_{1}^{2}r_{i}; 2 \le p_{i} \le \kappa .$$ Taking summation over $$ P_{i} $$, we have

10 $$\begin{aligned} \sum _{p_{i}=2}^{\kappa }I_{n}^{N}(v_{p_{1}})I_{n}^{N}(v_{p{i}})CONN_{I_{n}^{N}({\c{G}})}(v_{p_{1}},v_{p{i}})= & {} \sum _{p_{i}=2}^{\kappa }r_{1}^{2} r_{p_{i}}, \end{aligned}$$

for $$ v_{p_{2}},$$ is

11 $$\begin{aligned} \sum _{p_{i}=3}^{\kappa }I_{n}^{N}(v_{p_{2}})I_{n}^{N}(v_{p{i}})CONN_{I_{n}({\c{G}})}(v_{p_{2}},v_{p{i}})= & {} \sum _{p_{i}=3}^{\kappa }r_{2}^{2} r_{p_{i}}, \end{aligned}$$

for $$ v_{p_{3}} $$ is

12 $$\begin{aligned} \sum _{p_{i}=4}^{\kappa }I_{n}^{N}(v_{p_{3}})I_{n}^{N}(v_{p{i}})CONN_{I_{n}({\c{G}})}(v_{p_{3}},v_{p{i}})= & {} \sum _{p_{i}=4}^{\kappa }r_{3}^{2} r_{p_{i}}, \end{aligned}$$

and for $v_{\kappa -1}$ is

13 $$\begin{aligned} \sum _{p_{i}= \kappa }^{\kappa }I_{n}^{N}(v_{n-1})I_{n}^{N}(v_{p{i}})CONN_{I_{n}({\c{G}})}(v_{m-1},v_{p{i}})= & {} \sum _{p_{i}= \kappa }^{\kappa }r_{n-1}^{2} r_{p_{i}}. \end{aligned}$$

The result of combining the equations above is

14 $$\begin{aligned} I_{n}CI_{N}({\c{G}})= & {} \sum _{p_{i}=2}^{\kappa } r_{1}^{2} r_{p_{i}}+ \sum _{p_{i}=3}^{\kappa } r_{2}^{2} r_{i}+\sum _{p_{i}=4}^{\kappa } r_{3}^{2} r_{p_{i}}+\cdots + \sum _{p_{i}=\kappa }^{\kappa } r_{n-1}^{2} r_{p_{i}}\end{aligned}$$

15 $$\begin{aligned}= & {} \sum _{i=1}^{\kappa -1} r_{p_{i}}^{2} \sum _{p_{j}= p_{i}+1}^{\kappa } r_{p_{j}}. \end{aligned}$$

and Suppose $$v_{p_{1}} $$ is the vertex with least falsity-membership value $$s_{1}$$. Then, for a complete neutrosophic graph, $$ CONN_{F_{i}({\c{G}})}(u_{p},v_{p}) = F_{i}^{M}(u_{p},v_{p})\forall u_{p},v_{p} \in N^{*} $$, So, $$F_{i}^{M}(v_{p_{1}},v_{p_{i}})=s_{1}; 2 \le p_{i} \le \kappa $$ and hence, $$F_{i}^{N}(v_{p_{1}})F_{i}^{N}(v_{p_{i}}) CONN_{F_{i}({\c{G}})}(v_{p_{1}},v_{p_{i}})= s_{1}.s_{p_{i}}.s_{1} = s_{1}^{2}s_{p_{i}}; 2 \le p_{i} \le \kappa .$$

16 $$\begin{aligned} \sum _{p_{i}=2}^{\kappa }F_{i}^{N}(v_{p_{1}})F_{i}^{N}(v_{p{i}})CONN_{F_{i}({\c{G}})}(v_{p_{1}},v_{p_{i}})= & {} \sum _{p_{i}=2}^{\kappa }s_{1}^{2} s_{p_{i}}, \end{aligned}$$

for $$ v_{p_{2}},$$ is

17 $$\begin{aligned} \sum _{p_{i}=3}^{\kappa }F_{i}^{N}(v_{p_{2}})F_{i}^{N}(v_{p{i}})CONN_{F_{i}({\c{G}})}(v_{p_{2}},v_{p_{i}})= & {} \sum _{p_{i}=3}^{\kappa }s_{2}^{2} s_{p_{i}}, \end{aligned}$$

for $$ v_{p_{3}} $$ is

18 $$\begin{aligned} \sum _{p_{i}=4}^{\kappa }F_{i}^{N}(v_{p_{3}})F_{i}^{N}(v_{p{i}})CONN_{F_{i}({\c{G}})}(v_{p_{3}},v_{p{i}})= & {} \sum _{p_{i}=4}^{\kappa }s_{3}^{2} s_{p_{i}}, \end{aligned}$$

and for $v_{\kappa -1}$ is

19 $$\begin{aligned} \sum _{p_{i}=\kappa }^{\kappa }F_{i}^{N}(v_{\kappa -1})F_{i}^{N}(v_{p{i}})CONN_{F_{i}({\c{G}})}(v_{\kappa -1},v_{p_{i}})= & {} \sum _{p_{i}=\kappa }^{\kappa }s_{\kappa -1}^{2} s_{p_{i}}. \end{aligned}$$

By adding all the above equations, we get

20 $$\begin{aligned} F_{i}CI_{n}^{N}({\c{G}})= & {} \sum _{p_{i}=2}^{\kappa } s_{1}^{2} s_{p_{i}}+ \sum _{p_{i}=3}^{\kappa } s_{2}^{2} s_{p_{i}}+\sum _{p_{i}=4}^{\kappa } s_{3}^{2} s_{p_{i}}+\cdots + \sum _{p_{i}=\kappa }^{\kappa } s_{\kappa -1}^{2} s_{i} \end{aligned}$$

21 $$\begin{aligned}= & {} \sum _{i=1}^{\kappa -1} s_{p_{i}}^{2} \sum _{p_{j}= p_{i}+1}^{\kappa } s_{p_{j}}. \end{aligned}$$

Finally, Sum of all $CI_{N}s$,we {\c{G}}et

22 $$\begin{aligned} CI_{N}({\c{G}})= & {} T_{r}CI_{N}({\c{G}})+I_{n}CI_{N}({\c{G}})+F_{i}CI_{N}({\c{G}})\end{aligned}$$

23 $$\begin{aligned}= & {} \sum _{p_{i}=1}^{\kappa -1}t_{p_{i}}^{2} \sum _{p_{j}= p_{i}+1}^{\kappa } t_{p_{j}} + \sum _{i=1}^{\kappa -1}r_{p_{i}}^{2} \sum _{p_{j}= p_{i}+1}^{\kappa } r_{p_{j}}+ \sum _{p_{i}=1}^{\kappa -1}s_{p_{i}}^{2} \sum _{p_{j}=p{i}+1}^{\kappa } s_{p_{j}}. \end{aligned}$$

$$\square $$

### Example 5

**Figure 4:**
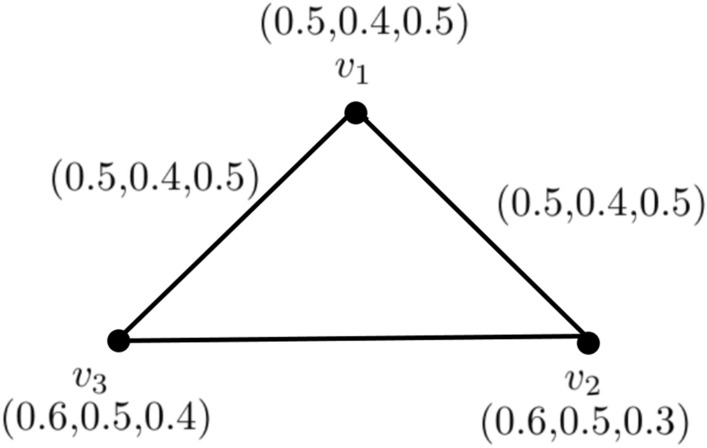
A complete neutrosophic graphv with $$ CI_{N}=1.012$$.

Figure 4 makes it clear that $k_{3}$ is an entirely neutrosophic graph. So,

$$\begin{aligned} T_{r}CI_{N}({\c{G}})= & {} \sum _{p_{i}=1}^{3}T_{r}^{N}(v_{p{i}})T_{r}^{N}(v_{p{j}})CONN_{T_{r}({\c{G}})}(v_{p{i}},v_{p{j}})\\= & {} (0.5)(0.6)(0.5)+(0.6)(0.6)(0.6)+(0.5)(0.6)(0.5)\\= & {} 0.516 \\ I_{n}CI_{N}({\c{G}})= & {} \sum _{p_{i}=1}^{3}I_{n}^{N}(v_{p{i}})I_{n}^{N}(v_{p{j}})CONN_{I_{n}({\c{G}})}(v_{p{i}},v_{p{j}})\\= & {} (0.4)(0.5)(0.4)+(0.5)(0.5)(0.5)+(0.4)(0.5)(0.4)\\= & {} 0.285 \\ F_{i}CI_{N}({\c{G}})= & {} \sum _{p_{i}=1}^{3}F_{i}^{N}(v_{p{i}})F_{i}^{N}(v_{p{j}})CONN_{F_{i}({\c{G}})}(v_{p{i}},v_{p{j}})\\= & {} (.5)(.4)(.5)+(.4)(.3)(.4)+(.5)(.3)(.5)\\= & {} 0.211.\\ CI_{N}({\c{G}})= & {} T_{r}CI_{N}({\c{G}})+I_{n}CI_{N}({\c{G}})+F_{i}CI_{N}({\c{G}}) \\= & {} 0.516+0.285+0.211 \\= & {} 1.012. \end{aligned}$$

Now, we use above theorem

$$\begin{aligned} \sum _{p_{i}=1}^{\kappa -1}t_{p_{i}}^{2} \sum _{p{j}=p{i}+1}^{\kappa } t_{p_{j}}= & {} \sum _{p_{i}=1}^{2}t_{p_{i}}^{2} \sum _{p_{j}=p_{i}+1}^{3} t_{p_{j}}\\= & {} t_{1}^{2}(t_{2}+t_{3})+t_{2}^{2}t_{3}\\= & {} (0.5)^{2}(0.6+0.6)+(0.6)^{2}(0.6)\\= & {} 0.516 \\ \sum _{p_{i}=1}^{\kappa -1}r_{p_{i}}^{2} \sum _{p_{j}= p_{i}+1}^{\kappa } r_{p_{j}}= & {} \sum _{p_{i}=1}^{2}r_{p_{i}}^{2} \sum _{p_{j}=p_{i}+1}^{3} r_{p_{j}}\\= & {} r_{1}^{2}(r_{2}+r_{3})+r_{2}^{2}r_{3}\\= & {} (0.4)^{2}(0.5+0.5)+(0.5)^{2}(0.5)\\= & {} 0.285 \\ \sum _{p_{i}=1}^{\kappa -1}s_{p_{i}}^{2} \sum _{p_{j}=p_{i}+1}^{\kappa } s_{p_{j}}= & {} \sum _{p_{i}=1}^{2}s_{p_{i}}^{2} \sum _{p_{j}=p_{i}+1}^{3} s_{p_{j}}\\= & {} s_{1}^{2}(s_{2}+s_{3})+s_{2}^{2}s_{3}\\= & {} (.5)^{2}(.3+.4)+(.3)^{2}(.4)=.211 \end{aligned}$$

Adding these three summations, we get

$$\begin{aligned} \sum _{p_{i}=1}^{\kappa -1}t_{p_{i}}^{2} \sum _{p_{j}=p_{i}+1}^{\kappa } t_{p_{j}} +\sum _{p_{i}=1}^{\kappa -1}r_{p_{i}}^{2} \sum _{p_{j}=p_{i}+1}^{\kappa } r_{p_{j}}+\sum _{p_{i}=1}^{\kappa -1}s_{p_{i}}^{2} \sum _{p_{j}= p_{i}+1}^{\kappa } s_{p_{j}}= & {} 0.516+0.285+0.211.\\= & {} 1.012 \end{aligned}$$

Hence, it is verified that

$$\begin{aligned}{} & {} CI_{N}({\c{G}}) = \sum _{p_{i}=1}^{\kappa -1}t_{p_{i}}^{2} \sum _{p_{j}=p_{i}+1}^{\kappa } t_{p_{j}}+\sum _{p_{i}=1}^{\kappa -1}r_{p_{i}}^{2} \sum _{p_{j}=p_{i}+1}^{\kappa } r_{p_{j}}+\sum _{p_{i}=1}^{\kappa -1}s_{p_{i}}^{2} \sum _{p_{j}= p_{i}+1}^{\kappa } s_{j}. \end{aligned}$$

## Connectivity index with edge and vertex deleted neutrosophic graphs

A vertex or an edge deletion may or may not have an impact on the $CI_{N}$. It is based on how the edge and vertex that must be omitted behave.

### Example 6

**Figure 5:**
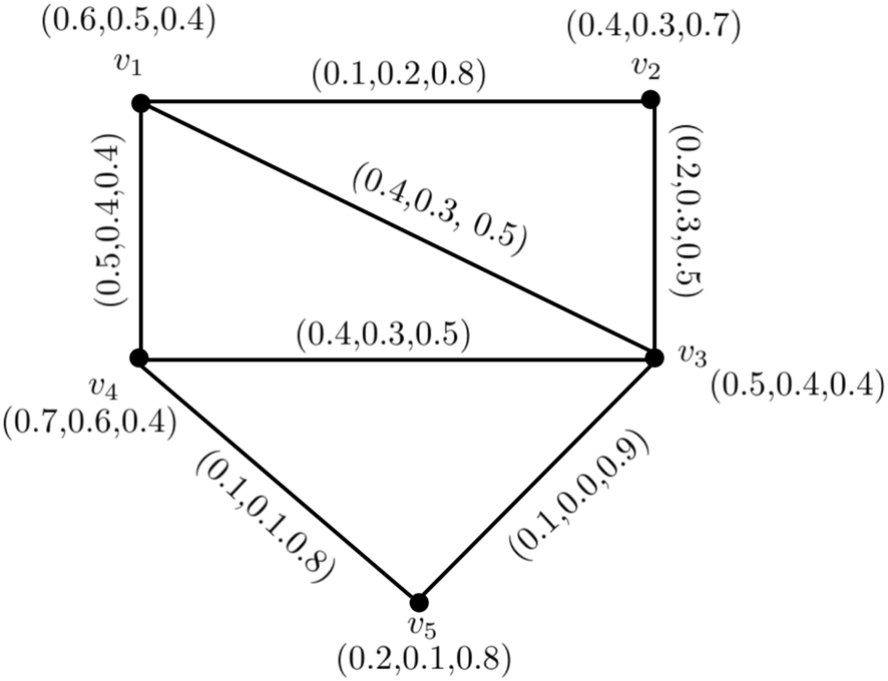
A neutrosophic graph $$ CI_{N}=2.529$$.

In Fig. 5, take $$ CI_{N} = 2.923$$ $${\c{G}}=(N,M)$$. Then $$(v_{p_{1}},v_{p_{4}}),(v_{p_{2}},v_{p_{3}}),(v_{p_{4}},v_{p_{5}})$$ are $$ \alpha $$-strong arcs,$$(v_{p_{1}},v_{p_{3}}),(v_{p_{3}},v_{p_{4}})$$ are $$ \beta $$-strong arcs and $$(v_{p_{1}},v_{p_{2}}),(v_{p_{3}},v_{p_{5}})$$ are $$ \gamma $$-strong arcs. Then,

$$\begin{aligned} T_{r}CI_{N}({\c{G}})&= \sum _{p_{i}=1}^{10}T_{r}^{N}(v_{p{i}})T_{r}^{N}(v_{p{j}})CONN_{T_{r}({\c{G}})}(v_{p{i}},v_{p{j}})\\&=(0.6)(0.4)(0.2)+(0.6)(0.5)(0.4)+(0.6)(0.7)(0.5)+(0.6)(0.2)(0.1)\\&\quad +(0.4)(0.5)(0.2)+(0.4)(0.7)(0.2)+(0.4)(0.2)(0.1)+(0.5)(0.7)(0.4)\\&\quad +(0.5)(0.2)(0.1)+(0.7)(0.2)(0.1)\\&=0.658 \end{aligned}$$

$$\begin{aligned} I_{n}CI_{N}({\c{G}})&= \sum _{p_{i}=1}^{10}I_{n}^{N}(v_{p{i}})I_{n}^{N}(v_{p{j}})CONN_{I_{n}({\c{G}})}(v_{p{i}},v_{p{j}})\\&=(0.5)(0.3)(0.3)+(0.5)(0.4)(0.3)+(0.5)(0.6)(0.4)+(0.5)(0.1)(0.1)\\&\quad +(0.3)(0.4)(0.3)+(0.3)(0.6)(0.3)+(0.3)(0.1)(0.1)+(0.4)(0.6)(0.3)\\&\quad +(0.4)(0.1)(0.1)+(0.6)(0.1)(0.1)\\&=0.405\\ F_{i}CI_{N}({\c{G}})&= \sum _{p_{i}=1}^{10}F_{i}^{N}(v_{p{i}})F_{i}^{N}(v_{p{j}})CONN_{F_{i}({\c{G}})}(v_{p{i}},v_{p{j}}) \\&= (.4)(.7)(.5)+(.4)(.4)(.5)+(.4)(.4)(.4)+(.4)(.8)(.8)+(.7)(.4)(.5)\\&\quad +(.7)(.4)(.5)+(.7)(.8)(.8)+(.4)(.4)(.5)+(.4)(.8)(.8)+(.4)(.8)(.8)\\&= 1.86 \end{aligned}$$

**Figure 6:**
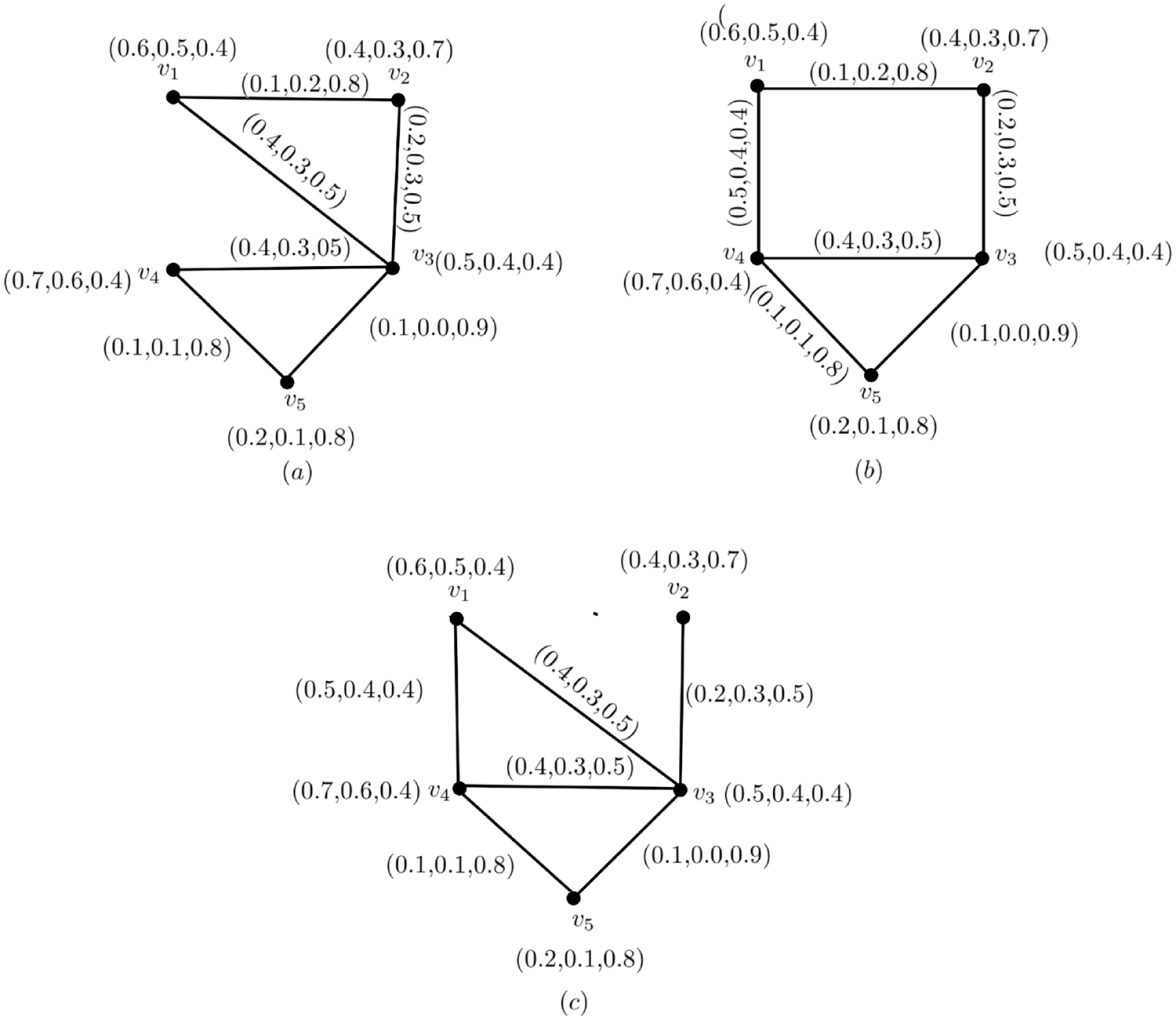
$${G}-(v_{p_{1}},v_{p_{4}}),{G}-(v_{p_{1}},v_{p_{3}})$$ and $$ {G}-(v_{p_{1}},v_{p_{2}})$$, (**a**)  $${G}-(v_{p_{1}},v_{p_{4}}).\, ({\textbf {b}})\, {G}-(v_{p_{1}},v_{p_{3}}), \, ({\textbf {c}})\, {G}-(v_{p_{1}},v_{p_{2}}) $$

$CI_{N}({\c{G}})= T_{r}CI_{N}({\c{G}})+I_{n}CI_{N}({\c{G}})+F_{i}CI_{N}({\c{G}})$ = 0.658+0.405+1.86 = 2.923. So, we have $CI_{N}({\c{G}}-(v_{p_{1}},v_{p_{4}}))= T_{r}CI_{N}({\c{G}}-(v_{p_{1}},v_{p_{4}}))+I_{n}CI_{N}({\c{G}}-(v_{p_{1}},v_{p_{4}}))+F_{i}CI_{N}({\c{G}}-(v_{p_{1}},v_{p_{4}})) = 0.448+0.285+1.796 = 2.529.$ Thus, $CI_{N}({\c{G}}-(v_{p_{1}},v_{p_{4}}))<CI_{N}({\c{G}})$, this implies $CI_{N}$ of ${\c{G}}$ have reduced by neglecting $\alpha$-strong edge $(v_{p_{1}},v_{p_{4}})$. The neutrosohic graph, ${\c{G}}-(v_{p_{1}},v_{p_{2}})$ Fig. 6, like Fig. 6a.

If we remove the $\beta$-strong edge $(v_{p_{1}},v_{p_{3}}),$ then every pair of vertices strength of connectivity is constant, ie.,

$$\begin{aligned} CONN_{T_{r}({\c{G}})}(v_{p{i}},v_{p{j}})&= CONN_{T_{r}({\c{G}})-(v_{p_{1}},v_{p_{3}})}(v_{p{i}},v_{p{j}}),\\ CONN_{I_{n}({\c{G}})}(v_{p{i}},v_{p{j}})&= CONN_{I_{n}({\c{G}})-(v_{p_{1}},v_{p_{3}})}(v_{p{i}},v_{p{j}})\\ CONN_{F_{i}({\c{G}})}(v_{p{i}},v_{p{j}})&= CONN_{F_{i}({\c{G}})-(v_{p_{1}},v_{p_{3}})}(v_{p{i}},v_{p{j}}), \end{aligned}$$

so $CI_{N}({\c{G}}-(v_{p_{1}},v_{p_{3}}))=CI_{N}({\c{G}}).$ The graph of ${\c{G}}-(v_{p_{1}},v_{p_{3}})$ according to Fig. 6b. Similarly, when we delete the $\gamma$-arc $(v_{p_{1}},v_{p_{2}})$, then both the $CI_{N}$ and the strength of connectivity between each pair of vertices remain constant. The graph of ${\c{G}}-(v_{p_{1}},v_{p_{2}})$ appears in Fig. 6c.

24 $$\begin{aligned} T_{r}CI_{N}({\c{G}}-(v_{p_{1}},v_{p_{4}}))&= \sum _{i=1}^{10}T_{r}^{N}(v_{p{i}})T_{r}^{N}(v_{p{j}})CONN_{T_{r}({\c{G}})-(v_{p_{1}},v_{p_{4}})}(v_{p{i}},v_{p{j}})= 0.448 \end{aligned}$$

25 $$\begin{aligned} I_{n}CI_{N}({\c{G}}-(v_{p_{1}},v_{p_{4}}))&= \sum _{i=1}^{10}I_{n}^{N}(v_{p{i}})I_{n}^{N}(v_{p{j}})CONN_{I_{n}({\c{G}})-(v_{p_{1}},v_{p_{4}})}(v_{p{i}},v_{p{j}})= 0.285 \end{aligned}$$

26 $$\begin{aligned} F_{i}CI_{N}({\c{G}}-(v_{p_{1}},v_{p_{4}}))&= \sum _{i=1}^{10}F_{i}^{N}(v_{p{i}})F_{r}^{N}(v_{p{j}})CONN_{F_{i}({\c{G}})-(v_{p_{1}},v_{p_{4}})}(v_{p{i}},v_{p{j}})= 1.790 \end{aligned}$$

### Theorem 1

Let *H* be the neutrosophic sub graph of a neutrosophic graph ${\c{G}}=(N,M)$ produced by taking away an edge $u_{p}v_{p} \in M_{{\c{G}}}$ from ${\c{G}}$. Then, $CI_{n}^{N}({\c{G}})> CI_{N}(H)$ or $(CI_{N})_{n}^{N}({\c{G}})< CI_{N}(H)$ iff $u_{p}v_{p}$ is a bridge.

### Proof

Consider $u_{p}v_{q}$ as a bridge. As stated in Definition 6, there exit $u_{p}$ and $v_{p}$ in a way that reduces the strength of their connection. So, We determine that $(CI_{N})_{n}^{N}({\c{G}})>CI_{N}(H)$ or $(CI_{N})_{n}^{N}({\c{G}})< CI_{N}(H)$. Conversely, let that $CI_{N}({\c{G}})>CI_{N}(H)$ or $CI_{N}({\c{G}})<CI_{N}(H)$ and give the following options some thought.

Case(a).: Consider, $u_{p}v_{p}$ is a $\gamma$-arc. Then, $CONN_{T_{r}({\c{G}})-u_{p}v_{p}}(u_{p},v_{p}) = CONN_{T_{r}({\c{G}})}(u_{p},v_{p}),$ $CONN_{I_{n}({\c{G}})-u_{p}v_{p}}(u_{p},v_{p}) = CONN_{I_{n}({\c{G}})}(u_{p},v_{p}),$ and $CONN_{F_{i}({\c{G}})-u_{p}v_{p}}(u_{p},v_{p})$ = $CONN_{F_{i}({\c{G}})}(u_{p},v_{p})$. So, we have $(T_{r}CI_{N})_{n}^{N}({\c{G}}) = T_{r}CI_{N}(H), (I_{n}CI_{N})_{n}({\c{G}}) = I_{n}CI_{N}(H)$ and $(F_{i}CI_{N})_{n}^{N}({\c{G}}) = F_{i}CI_{N}(H)$ and therefore, $(CI_{N})_{n}^{N}({\c{G}})= CI_{N}(H)$.
Case(b).: Let $u_{p}v_{p}$ as $\gamma$-strong arc. Then, $T_{r}^{M}(u_{p},v_{p}) = CONN_{T_{r}({\c{G}})-u_{p}v_{p}}(u_{p},v_{p}),$ $I_{n}^{M}(u_{p},v_{p}) = CONN_{I_{n}({\c{G}})-u_{p}v_{p}}(u_{p},v_{p})$ and $F_{i}^{M}(u_{p},v_{p}) = CONN_{F_{i}({\c{G}})-u_{p}v_{p}}(u_{p},v_{p})$. This implies that there is another $u_{p}-v_{p}$ path different from $u_{p}v_{p}$ edge. Therefore, the removal of the arc $u_{p}v_{p}$ will have no effect on the strength of connectedness between $u_{p}$ and $v_{p}$. So, $CI_{N}({\c{G}})=CI_{N}(H)$.
Case(c).: Now, let $u_{p}v_{p}$ be $\alpha$-strong edge. Then, $T_{r}^{M}(u_{p},v_{p})>CONN_{T_{r}({\c{G}})-uv}(u_{p},v_{p}),$ $I_{n}^{M}(u_{p},v_{p})>CONN_{I_{n}({\c{G}})-uv}(u_{p},v_{p})$ and $F_{i}^{M}(u_{p},v_{p})< CONN_{F_{i}({\c{G}})-u_{p}v_{p}}(u_{p},v_{p})$. Thus, the strongest path is $u_{p}v_{p}$ edge with strength equal to $(T_{r}^{M}(u_{p},v_{p}),I_{n}^{M}(u_{p},v_{p}),$ $F_{i}^{M}(u_{p},v_{p}))$. Then, clearly $CI_{N}({\c{G}})>CI_{N}(H),$ or $CI_{N}({\c{G}})<CI_{N}(H), T_{r}CI_{N}({\c{G}})-T_{r}CI_{N}(H)> I_{n}CI_{N}({\c{G}})-I_{n}CI_{N}(H) > F_{i}CI_{N}({\c{G}})-F_{i}CI_{N}(H)$ or $T_{r}CI_{N}({\c{G}})-T_{r}CI_{N}(H)< I_{n}CI_{N}({\c{G}})-I_{n}CI_{N}(H) < F_{i}CI_{N}({\c{G}})-F_{i}CI_{N}(H)$ since $\alpha$-strong edges are neutrosophic bridges. This implies that $u_{p}v_{p}$ is a bridge.

$$\square $$

### Remark 1

Let *H* be the neutrosophic sub graphof a neutrosophic graph {\c{G}}=(Ņ,M̧) formulated by removing an edge $u_{p}v_{p} \in M_{{\c{G}}}$ from ${\c{G}}$. Then, $CI_{N}({\c{G}})= CI_{N}(H) \Leftrightarrow u_{p}v_{p}$ is either $\beta$-strong or $\gamma$-edge.

### Remark 2

Consider the case when $u_{p}v_{p}$ is an edge of a neutrosophic graph {\c{G}}=(Ņ,M̧). Then, $CI_{N}({\c{G}})\ne CI_{N}({\c{G}}-u_{p}v_{p})$ if and only if a unique neutrosophic bridge of ${\c{G}}$ is $u_{p}v_{p}$.

### Theorem 2

Let ${\c{G}}_{1}=(N_{1},M_{1})$ and ${\c{G}}_{2} = (N_{2},M_{2})$ be the two isomorphic neutrosophic graphs. Then, $CI_{N}({\c{G}}_{1})= CI_{N}({\c{G}}_{2})$.

### Proof

Suppose that $${\c{G}}_{1}=( N_{1},M_{1})$$ and $$ {\c{G}}_{2} = (N_{2},M_{2})$$ are isomorphic neutrosophic graphs. Then, $$ \exists $$ is a mapping $$ h: N_{1} \rightarrow N_{2}$$ such that *h* is bijective and $$ T_{N_{1}} (u_{p_{i}})= T_{N_{2}}(h(u_{p_{i}})), I_{N_{1}}(u_{p_{i}})= I_{N_{2}}(h(u_{p_{i}})) $$ and $$ F_{N_{1}} (u_{p_{i}})= F_{N_{2}}(h(u_{p_{i}}))$$ for all $$ u_{p_{i}} \in N^{*} $$ as well as $$ T_{M_{1}} (u_{p_{i}},u_{p_{j}})= T_{M_{2}}(h(u_{p_{i}}),(u_{p_{j}})), I_{M_{1}}(u_{p_{i}},u_{p_{j}})= I_{M_{2}}(h(u_{p_{i}}),(u_{p_{j}}))$$ and $$ F_{M_{1}}(u_{p_{i}},u_{p_{j}})= F_{M_{2}}(h(u_{p_{i}}),(u_{p_{j}}))$$ for all $$ u_{p_{i}} \in M^{*} $$. As $${\c{G}}_{1}$$ and $${\c{G}}_{2}$$ isomorphic, then the strength of any strongest $$u_{p_{i}} - u_{p_{j}}$$ is equal to $$h(u_{p_{i}}) - h(u_{p_{j}})$$ in $${\c{G}}_{2}$$. Thus, $$u_{p},v_{p} \in N^{*}$$

27 $$\begin{aligned} CONN_{T_{r}({\c{G}}_{1})}(u_{p_{i}},u_{p_{j}})= & {} CONN_{T_{r}({\c{G}}_{2})}(h(u_{p_{i}}),h(u_{p_{j}})), \end{aligned}$$

28 $$\begin{aligned} CONN_{I_{n}({\c{G}}_{1})}(u_{p_{i}},u_{p_{j}})= & {} CONN_{I_{n}({\c{G}}_{2})}(h(u_{p_{i}}),h(u_{p_{j}})),\end{aligned}$$

29 $$\begin{aligned} CONN_{F_{i}({\c{G}}_{1})}(u_{p_{i}},u_{p_{j}})= & {} CONN_{F_{i}({\c{G}}_{2})}(h(u_{p_{i}}),h(u_{p_{j}})) \end{aligned}$$

So, we have

30 $$\begin{aligned} T_{r}CI_{N}({\c{G}}_{1})= & {} \sum _{u_{p_{i}},u_{p_{j}}\in V({\c{G}}_{1})}T_{N_{1}}(u_{p_{i}}) T_{N_{1}}(u_{p_{j}})CONN_{T({\c{G}}_{1})}(u_{p_{i}},u_{p_{j}}) \end{aligned}$$

31 $$\begin{aligned}= & {} \sum _{h(u_{p_{i}}),h(u_{p_{j}}) \in V({\c{G}}_{2})}T_{N_{2}}(h(u_{p_{i}})) T_{N_{2}}(h(u_{p_{j}})) CONN_{T({\c{G}}_{2})}(h(u_{p_{i}}),h(u_{p_{j}}))\end{aligned}$$

32 $$\begin{aligned}= & {} T_{r}CI_{N}({\c{G}}_{2})\end{aligned}$$

33 $$\begin{aligned} I_{n}CI_{N}({\c{G}}_{1})= & {} \sum _{u_{p_{i}},u_{p_{j}}\in V({\c{G}}_{1})}I_{N_{1}}(u_{p_{i}})I_{N_{1}}(u_{p_{j}})CONN_{I({\c{G}}_{1})}(u_{p_{i}},u_{p_{j}})\end{aligned}$$

34 $$\begin{aligned}= & {} \sum _{h(u_{p_{i}}),h(u_{p_{j}}) \in V({\c{G}}_{2})}I_{N_{2}}(h(u_{p_{i}}))I_{N_{2}}(h(u_{p_{j}})) CONN_{I({\c{G}}_{2})}(h(u_{p_{i}}),h(u_{p_{j}}))\end{aligned}$$

35 $$\begin{aligned}= & {} I_{n}CI_{N}({\c{G}}_{2})\end{aligned}$$

36 $$\begin{aligned} F_{i}CI_{N}({\c{G}}_{1})= & {} \sum _{u_{p_{i}},u_{p_{j}}\in V({\c{G}}_{1})}F_{N_{1}}(u_{p_{i}}) F_{N_{1}}(u_{p_{j}})CONN_{F({\c{G}}_{1})}(u_{p_{i}},u_{p_{j}})\end{aligned}$$

37 $$\begin{aligned}= & {} \sum _{h(u_{p_{i}}),h(u_{p_{j}}) \in V({\c{G}}_{2})}F_{N_{2}}(h(u_{p_{i}})) F_{N_{2}}(h(u_{p_{j}})) CONN_{F({\c{G}}_{2})}(h(u_{p_{i}}),h(u_{p_{j}})) \end{aligned}$$

38 $$\begin{aligned}= & {} F_{i}CI_{N}({\c{G}}_{2}). \end{aligned}$$

Thus, $T_{r}CI_{N}({\c{G}}_{1})+I_{n}CI_{N}({\c{G}}_{1})+F_{i}CI_{N}({\c{G}}_{1})=T_{r}CI_{N}({\c{G}}_{2})+I_{n}CI_{N}({\c{G}}_{2})+F_{i}CI_{N}({\c{G}}_{2}.$ This implies that $CI_{N}({\c{G}}_{1})= CI_{N}({\c{G}}_{2})$. $\square$

## Neutrosophic average connectivity index graph

The literature on intuitionistic fuzzy graphs contains the idea of average connection index. Therefore, the authors have presented this idea for neutrosophic graphs. The average flow of a network ensures its stability.

### Definition 12

The average $T_{r}$-connectivity index of ${\c{G}}$ is defined by

39 $$\begin{aligned} AT_{r}CI_{N}({\c{G}})&= \dfrac{1}{\left( {\begin{array}{c}\kappa \\ 2\end{array}}\right) }\sum _{u_{p},v_{p} \in N^{*}}T_{r}^{N}(u_{p})T_{r}^{N}(v_{p})CONN_{T_{r}({\c{G}})}(u_{p},v_{p}) \end{aligned}$$

the average $I_{n}$-connectivity index of ${\c{G}}$ is defined by

40 $$\begin{aligned} AI_{n}CI_{N}({\c{G}})&= \dfrac{1}{\left( {\begin{array}{c}\kappa \\ 2\end{array}}\right) }\sum _{u_{p},v_{p} \in N^{*}}I_{n}^{N}(u_{p})I_{n}^{N}(v_{p})CONN_{I_{n}({\c{G}})}(u_{p},v_{p}) \end{aligned}$$

the average $F_{i}$-connectivity index of ${\c{G}}$ is defined by

41 $$\begin{aligned} AF_{i}CI_{N}({\c{G}})&= \dfrac{1}{\left( {\begin{array}{c}\kappa \\ 2\end{array}}\right) }\sum _{u_{p},v_{p} \in N^{*}}F_{i}^{N}(u_{p})F_{i}^{N}(v_{p})CONN_{F_{i}({\c{G}})}(u_{p},v_{p}) \end{aligned}$$

where $CONN_{T_{r}({\c{G}})}(u_{p},v_{p})$ is the $T_{r}$-strength of connectedness,$CONN_{I_{n}^{N}({\c{G}})}(u_{p},v_{p})$ is the $I_{n}$-strength of connectedness and $CONN_{F_{i}({\c{G}})}(u_{p},v_{p})$ is the $F_{i}-$strength $u_{p} - v_{p}$.

### Definition 13

Let {\c{G}}=(Ņ,M̧) be a neutrosophic graph. Then, the average connectivity index of ${\c{G}}$ is defined to be the sum of average $T_{r}$-connectivity index, $I_{n}$-connectivity index and $F_{i}$-connectivity index of ${\c{G}}$,

42 $$\begin{aligned} ACI_{N}({\c{G}})&= \dfrac{1}{\left( {\begin{array}{c}\kappa \\ 2\end{array}}\right) }\sum _{u_{p},v_{p} \in N^{*}}T_{r}^{N}(u_{p})T_{r}^{N}(v_{p})CONN_{T_{r}({\c{G}})}(u_{p},v_{p})\nonumber \\&\quad + \dfrac{1}{\left( {\begin{array}{c}\kappa \\ 2\end{array}}\right) }\sum _{u_{p},v_{p} \in N^{*}}I_{n}^{N}(u_{p})I_{n}^{N}(v_{p})CONN_{I_{n}({\c{G}})}(u_{p},v_{p})\nonumber \\&\quad + \dfrac{1}{\left( {\begin{array}{c}\kappa \\ 2\end{array}}\right) }\sum _{u_{p},v_{p} \in N^{*}}F_{i}^{N}(u_{p})F_{i}^{N}(v_{p})CONN_{F_{i}({\c{G}})}(u_{p},v_{p}) \end{aligned}$$

43 $$\begin{aligned}&= AT_{r}CI_{N}({\c{G}})+AI_{n}CI_{N}({\c{G}})+AF_{i}CI_{N}({\c{G}}) \end{aligned}$$

where $CONN_{T_{r}({\c{G}})}(u_{p},v_{p})$ is the $T_{r}$-strength of connectedness,$CONN_{I_{n}({\c{G}})}(u_{p},v_{p})$ is the $I_{n}$-strength of connectedness and $CONN_{F_{i}({\c{G}})}(u_{p},v_{p})$ is the $F_{i}$-strength $u_{p} - v_{p}$.

### Example 7

**Figure 7:**
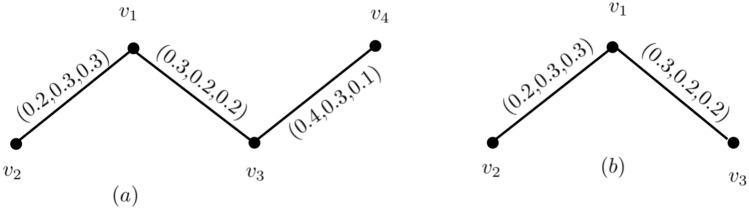
A neutrosophic graph ${\c{G}}-v_{p_{4}}$. (**a**) A neutrosophic graph with $CI_{N}=2.405.$ (**b**) ${\c{G}}-v_{p_{4}}$.

In Fig. 7, let {\c{G}}=(Ņ,M̧) be the neutrosophic graph with $$(T_{r}^{N}(v_{p}),I_{n}^{N}(v_{p}),F_{i}^{N}(v_{p})) = (.9,.9,.2) \forall v_{p} \in N^{*} $$. Then, we have $$(T_{r}CI_{N})_{n}^{N}({\c{G}})=1.215,(I_{n}CI_{N})_{n}^{N}({\c{G}})= 1.134, (F_{i}CI_{N})_{n}^{N}({\c{G}})= 0.056 $$ and $$ CI_{N} = 2.405$$.

From Example 7, $(CI_{N})_{n}^{N}({\c{G}})=2.405$ and the number of pair in ${\c{G}}$ is $$ \left( {\begin{array}{c}4\\ 6\end{array}}\right) \ = 6.$$ By average in {\c{G}}the $(T_{r}CI_{N})_{n}^{N}({\c{G}}), (I_{n}CI_{N})_{n}^{N}({\c{G}}), (F_{i}CI_{N})_{n}^{N}({\c{G}})$ and $(CI_{N})_{n}^{N}({\c{G}})$, we get

$$\begin{aligned} (AT_{r}CI_{N})_{n}^{N}({\c{G}})&= \dfrac{1}{6}(T_{r}CI_{N})_{n}^{N}({\c{G}}) = \dfrac{1}{6}(1.215)=0.2025,\\ (AI_{n}CI_{N})_{n}^{N}({\c{G}})&= \dfrac{1}{6}(I_{n}CI_{N})_{n}^{N}({\c{G}}) = \dfrac{1}{6}(1.134)= 0.189, \\ (AF_{i}CI_{N})_{n}^{N}({\c{G}})&= \dfrac{1}{6}(F_{i}CI_{N})_{n}^{N}({\c{G}}) = \dfrac{1}{6}(0.056)=0.0093 \\ (ACI_{N})_{n}^{N}({\c{G}})&= 0.2025+0.189+0.0093 = 0.4008. \end{aligned}$$

Now,consider ${\c{G}}-v_{p_{4}}$ and we have $T_{r}CI_{N}({\c{G}}-v_{p_{4}})= 0.567, I_{n}CI_{N}({\c{G}}-v_{p_{4}})= 0.648, F_{i}CI_{N}({\c{G}}-v_{p_{4}})= 0.036$ and $CI_{N}({\c{G}}-v_{p_{4}})=0.567+0.648+0.036 = 1.251$. On average in {\c{G}}them, we have

$$\begin{aligned} AT_{r}CI_{N}({\c{G}}-v_{p_{4}})= & {} \dfrac{ 0.567}{3}=0.188, \\ AI_{n}CI_{N}({\c{G}}-v_{p_{4}})= & {} \dfrac{0.648}{3}=0.216, \\ AF_{i}CI_{N}({\c{G}}-v_{p_{4}})= & {} \dfrac{0.036}{3}=0.012 \\ ACI_{N}({\c{G}}-v_{p_{4}})= & {} 0.188+0.216+0.012 = 0.416. \end{aligned}$$

By eliminating vertices, {\c{G}}’s total connectedness is enhanced $v_{p_{4}}$ from ${\c{G}}$.

**Figure 8:**
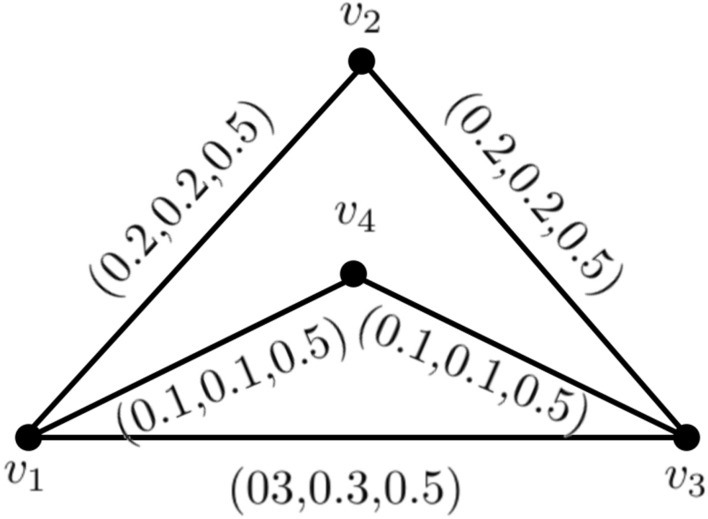
A neutrosophic graph with NCRN,NCEN and neutrosophic neutal nodes.

### Definition 14

Let ${\c{G}}=(N,M)$ be a neutrosohic graph and $u_{p},v_{p} \in N^{*}$. Then, $u_{p}$ is called a neutrosophic connectivity reducing node (*NCRN*) of ${\c{G}}$ if $ACI_{N}({\c{G}}-u_{p})< ACI_{N}({\c{G}}). u_{p}$ is termed as a neutrosophic connectivity enhancing node (*NCEN*) of ${\c{G}}$ if $ACI_{N}({\c{G}}-u_{p})> ACI_{N}({\c{G}}). u_{p}$ is said to be a neutrosophic neutral node of ${\c{G}}$ if $ACI_{N}({\c{G}}-u_{p})= ACI_{N}({\c{G}}).$

### Example 8

Consider the neutrosophic graphs shown in Fig. 8, We have taken here $$ ( T_{r}^{N}(u_{p}),I_{n}^{N}(u_{p}),F_{i}^{N}(u_{p})_=(0.5,0.5,0.5), \forall all u_{p}\in N^{*}. ACI_{N}({\c{G}})=0.2083, ACI_{N}({\c{G}}-v_{p_{1}})= 0.191,ACI_{N}({\c{G}}-v_{p_{2}})= 0.225,ACI_{N}({\c{G}}-v_{p_{3}})= 0.2083, ACI_{N}({\c{G}}-v_{p_{4}})= 0.2416$$. $$ ACI_{N}({\c{G}}-v_{p_{1}})<ACI_{N}({\c{G}}),ACI_{N}({\c{G}}-v_{p_{3}})= ACI_{N}({\c{G}}),$$ and $$ ACI_{N}({\c{G}}-v_{p_{2}}),ACI_{N}({\c{G}}-v_{p_{4}})> ACI_{N}({\c{G}})$$. Thus $$ v_{p_{1}}$$ is a *NCRN*,$$ v_{p_{3}}$$ is a neutral node, and $$ v_{p_{2}},v_{p_{4}}$$ are *NCEN*.

### Theorem 3

Let {\c{G}}=(Ņ,M̧) be a neutrosophic graph and $u_{p} \in N^{*}$ having $\vert N^{*}\vert \ge 3.$ Suppose $r = CI_{N}({\c{G}})/CI_{N}({\c{G}}-u_{p})$.u is a NCEN iff $r < \kappa /(\kappa -2)$. $u_{p}$ is a *NCRN* iff $r> \kappa /(\kappa -2). u_{p}$ is a neutrosophic neutral node iff $r= \kappa /(\kappa -2)$.

### Proof

Suppose $u_{p}$ is a neutrosophic neutral node. Then, $ACI_{N}({\c{G}}-u_{p}) = ACI_{N}({\c{G}})$. The $ACI_{N}$ is

44 $$\begin{aligned} \dfrac{1}{\left( {\begin{array}{c}\kappa \\ 2\end{array}}\right) }CI_{N}({\c{G}})&= \dfrac{1}{\left( {\begin{array}{c}\kappa -1\\ 2\end{array}}\right) }CI_{N}({\c{G}}-u_{p}). \end{aligned}$$

From here, we get

45 $$\begin{aligned} \dfrac{(CI_{N})_{m}^{N}({\c{G}})}{CI_{N}({\c{G}}-u_{p})}&= \dfrac{\left( {\begin{array}{c}\kappa \\ 2\end{array}}\right) }{\left( {\begin{array}{c}\kappa -1\\ 2\end{array}}\right) } \end{aligned}$$

46 $$\begin{aligned}&= \dfrac{\kappa (\kappa -1)/2}{(\kappa -1)(\kappa -2)/2} \end{aligned}$$

47 $$\begin{aligned}&= \dfrac{\kappa }{\kappa -2}. \end{aligned}$$

$$\square $$

### Theorem 4

Suppose {\c{G}}=(Ņ,M̧) be a neutrosophic graph with $\vert N^{*}\vert \ge 3.$ If $w_{p} \in N^{*}$ is an end vertex of ${\c{G}}$, let $l=\sum _{u_{p} \in N^{*}-{w_{p}}}CONN_{T_{r}({\c{G}})}(u_{p},w_{p})+\sum _{u_{p} \in N^{*}-{w_{p}}}CONN_{I_{n}({\c{G}})}(u_{p},w_{p})+\sum _{u_{p} \in N^{*}-{w_{p}}}CONN_{F_{i}({\c{G}})}(u_{p},w_{p})$. Then,

1. $w_{p}$ is a NCEN if $l <(2/(\kappa -2))CI_{N}({\c{G}}-w_{p})$
2. $w_{p}$ is a NCRN if $l >(2/(\kappa -2))CI_{N}({\c{G}}-w_{p})$
3. $w_{p}$ is a neutrosophic neutral node if $l = (2/(\kappa -2))CI_{N}({\c{G}}-w_{p})$

### Proof

Supoose $w_{p}$ be a neutrosophic neutral node. Then, $ACI_{N}({\c{G}}-w_{p}) = ACI_{N}({\c{G}}).$ We see that

48 $$\begin{aligned} CI_{N}({\c{G}})= & {} CI_{N}({\c{G}}-w_{p})+ \sum _{u \in N^{*}-{w}}CONN_{T_{r}({\c{G}})}(u_{p},w_{p})\nonumber \\{} & {} +\sum _{u_{p} \in N^{*}-{w_{p}}}CONN_{I({\c{G}})}(u_{p},w_{p})+\sum _{u_{p} \in N^{*}-{w_{p}}}CONN_{F_{i}({\c{G}})}(u_{p},w_{p})\end{aligned}$$

49 $$\begin{aligned} CI_{N}({\c{G}})= & {} CI_{N}({\c{G}}-w_{p})+l,\end{aligned}$$

50 $$\begin{aligned} l= & {} CI_{N}({\c{G}})-CI_{N}({\c{G}}-w_{p}) \end{aligned}$$

51 $$\begin{aligned} \dfrac{1}{\left( {\begin{array}{c}\kappa \\ 2\end{array}}\right) }l= & {} \dfrac{1}{\left( {\begin{array}{c}\kappa \\ 2\end{array}}\right) }CI_{N}({\c{G}})-\dfrac{1}{\left( {\begin{array}{c}\kappa \\ 2\end{array}}\right) }CI_{N}({\c{G}}-w_{p}) \end{aligned}$$

52 $$\begin{aligned}= & {} \dfrac{1}{\left( {\begin{array}{c}\kappa -1\\ 2\end{array}}\right) }CI_{N}({\c{G}}-w_{p})-\dfrac{1}{\left( {\begin{array}{c}\kappa \\ 2\end{array}}\right) }CI_{N}({\c{G}}-w_{p}) \end{aligned}$$

53 $$\begin{aligned}= & {} CI_{N}({\c{G}}-w_{p})\left[ \dfrac{1}{\left( {\begin{array}{c}\kappa -1\\ 2\end{array}}\right) }-\dfrac{1}{\left( {\begin{array}{c}\kappa \\ 2\end{array}}\right) } \right] , \end{aligned}$$

54 $$\begin{aligned} l= & {} CI_{N}({\c{G}}-w_{p}) \left[ \dfrac{\left( {\begin{array}{c}\kappa \\ 2\end{array}}\right) }{\left( {\begin{array}{c}\kappa -1\\ 2\end{array}}\right) }-1 \right] \end{aligned}$$

55 $$\begin{aligned}= & {} \dfrac{2}{\kappa -2} CI_{N}({\c{G}}-w_{p}). \end{aligned}$$

$$\square $$

## Application of neutrosophic graph with transport network flow

**Figure 9:**
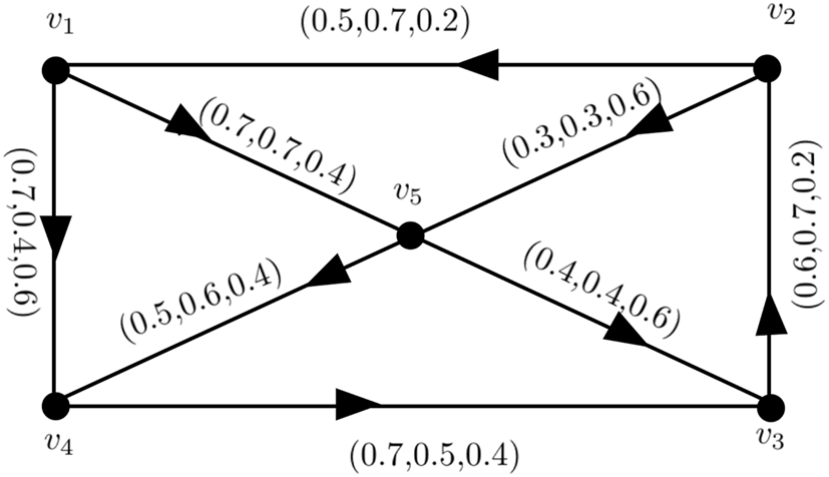
various connection indexes when a vertex is removed.

Take a neutrosophic directed network ${\c{G}}$, with traffic flow, as seen in Fig. 9 Assume $T_{r}^{N}(v_{p}),I_{n}^{N}(v_{p}),F_{i}^{N}(v_{p}))=(0.8,0.8,0.2)$ for all $v_{p} \in V({\c{G}}).$ Undirected neutrosophic graph and directed neutrosophic graph have identical connectivity. Both the directed and undirected neutrosophic graphs have comparable connectedness. Therefore, these notions may be expanded to directed neutrosophic graphs. The junctions at the vertices include correct, indeterminate and incorrect metrics for vehicles. The weights of the edges, which stand for the roadways that connect two junctions, represent the amount of vehicles carrying correct, indeterminant and incorrect information. Now, the authors will talk about certain network flow connection aspects.

First, the corresponding $T_{r}$ connectivity matrix will be identified; $TM({\c{G}})$ is the directed neutrosophic graphs.
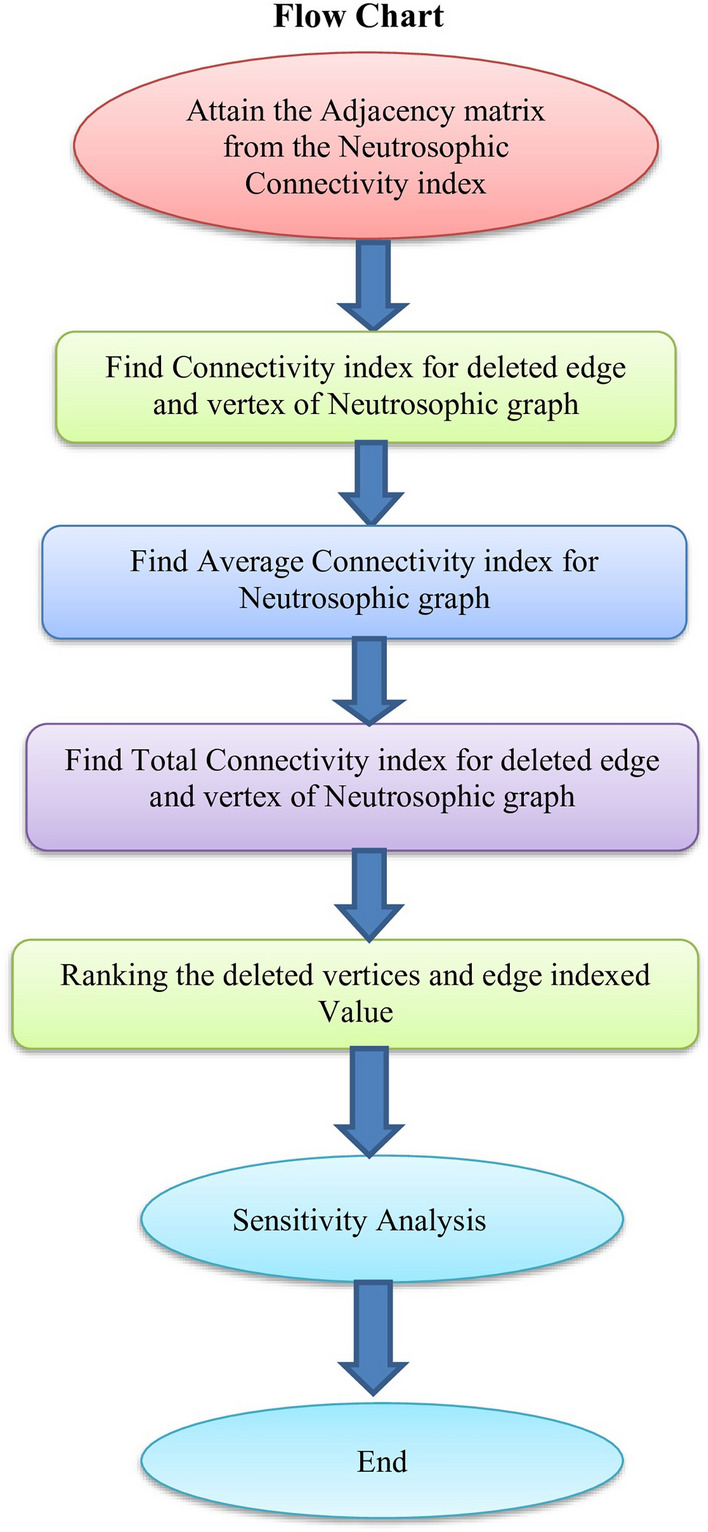

$$\begin{aligned} TM({\c{G}})= \begin{bmatrix} 0 &{} 0.4 &{} 0.7 &{} 0.7 &{} 0.7 \\ 0.5 &{} 0 &{} 0.5 &{} 0.5 &{} 0.5 \\ 0.5 &{} 0.6 &{} 0 &{} 0.5 &{} 0.5 \\ 0.5 &{} 0.6 &{} 0.7 &{} 0 &{} 0.5 \\ 0.4 &{} 0.4 &{} 0.4 &{} 0.5 &{} 0 \end{bmatrix} \end{aligned}$$

The matrix above is not symmetric since the graph is directed. We must thus add up every element of the matrix. Thus, $$ T_{r}CI_{N}({\c{G}})= 6.784, AT_{r}CI_{N}({\c{G}})= 0.6784.$$ Now, the associated $$ I_{n}$$-connectivity matrix $$IM({\c{G}})$$ is given as follows

$$\begin{aligned} IM({\c{G}})= \begin{bmatrix} 0 &{} 0.4 &{} 0.5 &{} 0.4 &{} 0.7 \\ 0.7 &{} 0 &{} 0.4 &{} 0.6 &{} 0.7 \\ 0.7 &{} 0.7 &{} 0 &{} 0.6 &{} 0.7 \\ 0.5 &{} 0.5 &{} 0.5 &{} 0 &{} 0.5 \\ 0.4 &{} 0.4 &{} 0.4 &{} 0.6 &{} 0 \end{bmatrix} \end{aligned}$$

cumulative each component of the matrix. Thus, $I_{n}CI_{N}({\c{G}})= 6.976, AI_{n}CI_{N}({\c{G}})= 0.6976.$ The corresponding $FM({\c{G}})$ matrix for $F_{i}$-connectivity is now stated.

$$\begin{aligned} FM({\c{G}})= \begin{bmatrix} 0 &{} 0.6 &{} 0.4 &{} 0.6 &{} 0.6 \\ 0.2 &{} 0 &{} 0.6 &{} 0.4 &{} 0.6 \\ 0.2 &{} 0.2 &{} 0 &{} 0.4 &{} 0.4 \\ 0.4 &{} 0.4 &{} 0.4 &{} 0 &{} 0.4 \\ 0.6 &{} 0.6 &{} 0.6 &{} 0.6 &{} 0 \end{bmatrix} \end{aligned}$$

By summing up all of $FM({\c{G}})$ entries, we get $F_{i}CI_{N}({\c{G}})= 3.600, AF_{i}CI_{N}({\c{G}})= 0.360.$ Thus, $ACI_{N}({\c{G}}) = AT_{r}CI_{N}({\c{G}})+AI_{n}CI_{N}({\c{G}})+AF_{i}CI_{N}({\c{G}}) = 0.6784+0.6976+0.360 = 1.736$ Consider ${\c{G}}-v_{p_{5}}$. Moreover, it is a directed neutrosophic graph. The matrices $TM({\c{G}}-v_{p_{5}}), IM({\c{G}}-v_{p_{5}})$ and $FM({\c{G}}-v_{p_{5}})$ are given by

$$\begin{aligned}{} & {} TM({\c{G}}-v_{p_{5}})= \begin{bmatrix} 0 &{} 0.6 &{} 0.7 &{} 0.7 \\ 0.5 &{} 0 &{} 0.5 &{} 0.5 \\ 0.5 &{} 0.6 &{} 0 &{} 0.5 \\ 0.5 &{} 0.6 &{} 0.7 &{} 0 \end{bmatrix}\\{} & {} IM({\c{G}}-v_{p_{5}})= \begin{bmatrix} 0 &{} 0.4 &{} 0.4 &{} 0.4 \\ 0.7 &{} 0 &{} 0.4 &{} 0.4 \\ 0.7 &{} 0.7 &{} 0 &{} 0.4 \\ 0.5 &{} 0.5 &{} 0.5 &{} 0 \end{bmatrix}\\{} & {} FM({\c{G}}-v_{p_{5}})= \begin{bmatrix} 0 &{} 0.6 &{} 0.6 &{} 0.6 \\ 0.2 &{} 0 &{} 0.6 &{} 0.6 \\ 0.2 &{} 0.2 &{} 0 &{} 0.6 \\ 0.4 &{} 0.4 &{} 0.4 &{} 0 \end{bmatrix} \end{aligned}$$

by calculation we have

56 $$\begin{aligned} T_{r}CI_{N}({\c{G}}-v_{p_{5}})&=4.416,AT_{r}CI_{N}({\c{G}}-v_{p_{5}})= 4.416/6 = 0.736 \end{aligned}$$

57 $$\begin{aligned} I_{n}CI_{N}({\c{G}}-v_{p_{5}})&= 3.84,AI_{n}CI_{N}({\c{G}}-v_{p_{5}})= 3.84/6 = 0.464 \end{aligned}$$

58 $$\begin{aligned} F_{i}CI_{N}({\c{G}}-v_{p_{5}})&= 0.216,AF_{i}CI_{N}({\c{G}}-v_{p_{5}})= 0.216/6 = 0.036. \end{aligned}$$

Thus

59 $$\begin{aligned} ACI_{N}({\c{G}}-v_{p_{5}})&=AT_{r}CI_{N}({\c{G}}-v_{p_{5}})+AI_{n}CI_{N}({\c{G}}-v_{p_{5}})+AF_{i}CI_{N}({\c{G}}-v_{p_{5}}) \end{aligned}$$

60 $$\begin{aligned}&= 0.736+0.464+0.036 = 1.412. \end{aligned}$$

As $ACI_{N}({\c{G}}-v_{p_{5}}) < ACI_{N}({\c{G}})$, which implies that $v_{p_{5}}$ is *NCRN*. After that, we consider ${\c{G}}-v_{p_{5}}$. The matrices $TM({\c{G}}-v_{p_{1}}),IM({\c{G}}-v_{p_{1}})$ and $FM({\c{G}}-v_{p_{1}})$ its given by

$$\begin{aligned}{} & {} TM({\c{G}}-v_{p_{1}})= \begin{bmatrix} 0 &{} 0.3 &{} 0.3 &{} 0.3 \\ 0.6 &{} 0 &{} 0.3 &{} 0.3 \\ 0.6 &{} 0.7 &{} 0 &{} 0.3 \\ 0.5 &{} 0.5 &{} 0.5 &{} 0 \end{bmatrix}\\{} & {} IM({\c{G}}-v_{p_{1}})= \begin{bmatrix} 0 &{} 0.3 &{} 0.3 &{} 0.3 \\ 0.7 &{} 0 &{} 0.3 &{} 0.3 \\ 0.5 &{} 0.5 &{} 0 &{} 0.3 \\ 0.5 &{} 0.5 &{} 0.6 &{} 0 \end{bmatrix}\\{} & {} FM({\c{G}}-v_{p_{1}})= \begin{bmatrix} 0 &{} 0.6 &{} 0.6 &{} 0.6 \\ 0.2 &{} 0 &{} 0.6 &{} 0.6 \\ 0.4 &{} 0.4 &{} 0 &{} 0.6 \\ 0.4 &{} 0.4 &{} 0.4 &{} 0 \end{bmatrix} \end{aligned}$$

we have

61 $$\begin{aligned} T_{r}CI_{N}({\c{G}}-v_{p_{1}})&=3.328,AT_{r}CI_{N}({\c{G}}-v_{p_{1}})= 3.328/6 = 0.555 \end{aligned}$$

62 $$\begin{aligned} I_{n}CI_{N}({\c{G}}-v_{p_{1}})&= 3.264,AI_{n}CI_{N}({\c{G}}-v_{p_{1}})= 3.264/6 = 0.544 \end{aligned}$$

63 $$\begin{aligned} F_{i}CI_{N}({\c{G}}-v_{p_{1}})&= 2.32,AF_{i}CI_{N}({\c{G}}-v_{p_{1}})= 2.32/6 = 0.3867. \end{aligned}$$

Thus

64 $$\begin{aligned} ACI_{N}({\c{G}}-v_{p_{1}})&=AT_{r}CI_{N}({\c{G}}-v_{p_{1}})+AI_{n}CI_{N}({\c{G}}-v_{p_{1}})+AF_{i}CI_{N}({\c{G}}-v_{p_{1}}) \end{aligned}$$

65 $$\begin{aligned}&= 0.555+0.544+0.3867 = 1.4857. \end{aligned}$$

As $ACI_{N}({\c{G}}-v_{p_{1}}) < ACI_{N}({\c{G}})$, which implies that $v_{p_{1}}$ is *NCRN*. After that, we consider ${\c{G}}-v_{p_{2}}$. The matrices $TM({\c{G}}-v_{p_{2}}),IM({\c{G}}-v_{p_{2}})$ and $FM({\c{G}}-v_{p_{2}})$ its given by

$$\begin{aligned}{} & {} TM({\c{G}}-v_{p_{2}})= \begin{bmatrix} 0 &{} 0.7 &{} 0.7 &{} 0.7 \\ 0 &{} 0 &{} 0 &{} 0 \\ 0 &{} 0.7 &{} 0 &{} 0 \\ 0 &{} 0.5 &{} 0.5 &{} 0 \end{bmatrix}\\{} & {} IM({\c{G}}-v_{p_{2}})= \begin{bmatrix} 0 &{} 0.4 &{} 0.6 &{} 0.7 \\ 0 &{} 0 &{} 0 &{} 0 \\ 0 &{} 0.5 &{} 0 &{} 0 \\ 0.5 &{} 0.5 &{} 0.6 &{} 0 \end{bmatrix} \\{} & {} FM({\c{G}}-v_{p_{2}})= \begin{bmatrix} 0 &{} 0.6 &{} 0.6 &{} 0.4 \\ 0 &{} 0 &{} 0 &{} 0 \\ 0 &{} 0.4 &{} 0 &{} 0 \\ 0 &{} 0.4 &{} 0.4 &{} 0 \end{bmatrix} \end{aligned}$$

we have

66 $$\begin{aligned} T_{r}CI_{N}({\c{G}}-v_{p_{2}})&=2.432,AT_{r}CI_{N}({\c{G}}-v_{p_{2}})=2.432/6 =0.4053 \end{aligned}$$

67 $$\begin{aligned} I_{n}CI_{N}({\c{G}}-v_{p_{2}})&= 2.112,AI_{n}CI_{N}({\c{G}}-v_{p_{2}})=2.112/6 =0.352 \end{aligned}$$

68 $$\begin{aligned} F_{i}CI_{N}({\c{G}}-v_{p_{2}})&= 1.12,AF_{i}CI_{N}({\c{G}}-v_{p_{2}})=1.12/6 =0.1867. \end{aligned}$$

Thus

69 $$\begin{aligned} ACI_{N}({\c{G}}-v_{p_{2}})&= AT_{r}CI_{N}({\c{G}}-v_{p_{2}})+AI_{n}CI_{N}({\c{G}}-v_{p_{2}})+AF_{i}CI_{N}({\c{G}}-v_{p_{2}}) \end{aligned}$$

70 $$\begin{aligned}&= 0.4053+0.352+0.1867= 0.9444. \end{aligned}$$

As $ACI_{N}({\c{G}}-v_{p_{2}}) < ACI_{N}({\c{G}})$, which implies that $v_{p_{2}}$ is *NCRN*. Now we consider ${\c{G}}-v_{p_{3}}$. The matrices $TM({\c{G}}-v_{p_{3}}),IM({\c{G}}-v_{p_{3}})$and$FM({\c{G}}-v_{p_{3}})$its given by

$$\begin{aligned}{} & {} TM({\c{G}}-v_{p_{3}})= \begin{bmatrix} 0 &{} 0 &{} 0.7 &{} 0.7 \\ 0.5 &{} 0 &{} 0.5 &{} 0.5 \\ 0 &{} 0 &{} 0 &{} 0 \\ 0 &{} 0 &{} 0.5 &{} 0 \end{bmatrix}\\{} & {} IM({\c{G}}-v_{p_{3}})= \begin{bmatrix} 0 &{} 0 &{} 0.4 &{} 0.7 \\ 0 &{} 0 &{} 0.6 &{} 0.7 \\ 0 &{} 0 &{} 0 &{} 0 \\ 0.5 &{} 0 &{} 0.6 &{} 0 \end{bmatrix}\\{} & {} FM({\c{G}}-v_{p_{3}})= \begin{bmatrix} 0 &{} 0 &{} 0.6 &{} 0.4 \\ 0.2 &{} 0 &{} 0.4 &{} 0.4 \\ 0 &{} 0 &{} 0 &{} 0 \\ 0 &{} 0 &{} 0.4 &{} 0 \end{bmatrix} \end{aligned}$$

we have

71 $$\begin{aligned} T_{r}CI_{N}({\c{G}}-v_{p_{3}})=2.176,AT_{r}CI_{N}({\c{G}}-v_{p_{3}})= 2.1762/6 =0.3627 \end{aligned}$$

72 $$\begin{aligned} I_{n}CI_{N}({\c{G}}-v_{p_{3}})=2.368,AI_{n}CI_{N}({\c{G}}-v_{p_{3}})= 2.368/6 =0.3947 \end{aligned}$$

73 $$\begin{aligned} F_{i}CI_{N}({\c{G}}-v_{p_{3}})=0.096,AF_{i}CI_{N}({\c{G}}-v_{p_{3}})= 0.096/6 =0.016. \end{aligned}$$

Thus

74 $$\begin{aligned} ACI_{N}({\c{G}}-v_{p_{3}})&=AT_{r}CI_{N}({\c{G}}-v_{p_{3}})+AI_{n}CI_{N}({\c{G}}-v_{p_{3}})+AF_{i}CI_{N}({\c{G}}-v_{p_{3}}) \end{aligned}$$

75 $$\begin{aligned}&= 0.3627+0.3947+0.016=0.7734. \end{aligned}$$

As $ACI_{N}({\c{G}}-v_{p_{3}}) < ACI_{N}({\c{G}})$, which implies that $v_{p_{3}}$ is *NCRN*. Now we consider ${\c{G}}-v_{p_{4}}$. The matrices $TM({\c{G}}-v_{p_{4}}), IM({\c{G}}-v_{p_{4}})$ and $FM({\c{G}}-v_{p_{4}})$ its given by

$$\begin{aligned}{} & {} TM({\c{G}}-v_{p_{4}})= \begin{bmatrix} 0 &{} 0.4 &{} 0.4 &{} 0.7 \\ 0.5 &{} 0 &{} 0.4 &{} 0.5 \\ 0.5 &{} 0.6 &{} 0 &{} 0.5 \\ 0.4 &{} 0.4 &{} 0.4 &{} 0 \end{bmatrix}\\{} & {} IM({\c{G}}-v_{p_{4}})= \begin{bmatrix} 0 &{} 0.4 &{} 0.4 &{} 0.7 \\ 0.7 &{} 0 &{} 0.4 &{} 0.7 \\ 0.7 &{} 0.7 &{} 0 &{} 0.7 \\ 0.7 &{} 0.4 &{} 0.4 &{} 0 \end{bmatrix}\\{} & {} FM({\c{G}}-v_{p_{4}})= \begin{bmatrix} 0 &{} 0.6 &{} 0.6 &{} 0.4 \\ 0.2 &{} 0 &{} 0.6 &{} 0.4 \\ 0.2 &{} 0.2 &{} 0 &{} 0.4 \\ 0.6 &{} 0.6 &{} 0.6 &{} 0 \end{bmatrix} \end{aligned}$$

we have

76 $$\begin{aligned} T_{r}CI_{N}({\c{G}}-v_{p_{4}})&=3.648,AT_{r}CI_{N}({\c{G}}-v_{p_{4}})= 3.648/6 = 0.608 \end{aligned}$$

77 $$\begin{aligned} I_{n}CI_{N}({\c{G}}-v_{p_{4}})&= 4.416,AI_{n}CI_{N}({\c{G}}-v_{p_{4}})= 4.416/6 = 0.736 \end{aligned}$$

78 $$\begin{aligned} F_{i}CI_{N}({\c{G}}-v_{p_{4}})&= 0.216,AF_{i}CI_{N}({\c{G}}-v_{p_{4}})= 0.216/6 =.036. \end{aligned}$$

Thus

79 $$\begin{aligned} ACI_{N}({\c{G}}-v_{p_{4}})&= AT_{r}CI_{N}({\c{G}}-v_{p_{4}})+AI_{n}CI_{N}({\c{G}}-v_{p_{4}})+AF_{i}CI_{N}({\c{G}}-v_{p_{4}})\end{aligned}$$

80 $$\begin{aligned}&= 0.608+0.736+0.036 = 1.38. \end{aligned}$$

As $ACI_{N}({\c{G}}-v_{p_{4}}) < ACI_{N}({\c{G}})$, which implies that $v_{p_{4}}$ is *NCRN*.

**Table 1:** Average connectivity indexes .

${\c{G}}-v_{p{i}}$	$ACI_{N}({\c{G}}-v_{p{i}})$	$\vert ACI_{N}({\c{G}})-ACI_{N}({\c{G}}-v_{p{i}}) \vert$
${\c{G}}-v_{p_{1}}$	1.4857	0.2503
${\c{G}}-v_{p_{2}}$	0.944	0.792
${\c{G}}-v_{p_{3}}$	0.7734	0.9626
${\c{G}}-v_{p_{4}}$	1.38	0.356

So, the removal of junction $v_{p_{1}}$ increases the average connectivity amongst the other junctions. Table 1show that there is a small difference between $ACI_{N}({\c{G}})$ and $ACI_{N}({\c{G}}-v_{p_{1}})$. So, the removal of $v_{p_{1}}$ has too much effect on the network. It is also seen that the difference between $ACI_{N}({\c{G}})$ and $ACI_{N}({\c{G}}-v_{p_{3}})$ higher than the other differences, so the removal of $v_{p_{3}}$ has maximum negative effects on the connectivity. The removal of $ACI_{N}({\c{G}}-v_{p_{2}})$ or $ACI_{N}({\c{G}}-v_{p_{4}})$ may have indeterminant effects on the traffic network flow.

**Table 2:** Connectivity indexes .

${\c{G}}-v_{p{i}}$	$CI_{N}({\c{G}}-v_{p{i}})$	$\vert CI_{N}({\c{G}})-CI_{N}({\c{G}}-v_{p{i}}) \vert$
${\c{G}}-v_{p_{1}}$	8.912	2.128
${\c{G}}-v_{p_{2}}$	5.664	1.12
${\c{G}}-v_{p_{3}}$	4.64	2.144
${\c{G}}-v_{p_{4}}$	8.288	1.496

As $CI_{N}({\c{G}}-v_{p_{2}}) < CI_{N}({\c{G}})$, which implies that $v_{p_{2}}$ is *NCRN*. So, the removal of junction $v_{p_{2}}$ increases the average connectivity amongst the other junctions. Table 2 show that there is a small difference between $CI_{N}({\c{G}})$ and $CI_{N}({\c{G}}-v_{p_{2}})$. So, the removal of $v_{p_{2}}$ has too much effect on the network.

It is also seen that the difference between $CI_{N}({\c{G}})$ and $CI_{N}({\c{G}}-v_{p_{3}})$ higher than the other differences, so the removal of $v_{p_{3}}$ has maximum negative effects on the connectivity. The removal of $CI_{N}({\c{G}}-v_{p_{1}})$ or $CI_{N}({\c{G}}-v_{p_{4}})$ may have indeterminant effects on the traffic network flow.

## Real life application

### Highway system and computer network

Accident rates are rising daily as a result of the heavy traffic on the roadways. The government should make considerable efforts to reduce the number of traffic accidents in order to reduce these incidents. To address this issue, a visual representation of neutrosophic graphs is offered here. The average connection indices between each pair of vertices in neutrosophic networks may be calculated to achieve this. The roads with the highest average connection index carry the most traffic and are the main sites of traffic accidents. To reduce accidents on certain roads, the government can build speed bumps, speed breakers, and deploy additional traffic wardens.

The exchange of data between computers in a network of several computers. The top performing computer or computers in a network that share the most data with all other computers in the network must be identified. The average connection indices between each pair of computers in a network may be computed to do this. The necessary computers for sending the most data to all the other computers in a network will be the pair of computers with the highest average connection indices.

## Sensitivity analysis

Sensitivity analysis shows that how various sources of uncertainty in a mathematical model contribute to the model’s overall uncertainty. This technique is used within specific boundaries that depend on one or more input variables. Based on the numerical results, we now calculate the corresponding outputs for changing input parameters one by one. Here, we make an attempt to compute the sensitivity analysis by changing the weights of the decision-makers within a certain range and observed the ranking order under different operators like connectivity index and Average connectivity index respectively as shown below in Table 1. From the Table 1, we observed that the removal of $v_{p_1}$ has too much effect on the network and the removal of $v_{p_3}$ has maximum negative effects on the connectivity. The $v_{p_2}$ and $v_{p_4}$ may have indeterminate effects on the traffic network flow. From the Table 2, we observed that the removal of $v_{p_2}$ has too much effect on the network and the removal of $v_{p_3}$ has maximum negative effects on the connectivity. The $v_{p_1}$ and $v_{p_4}$ may have indeterminate effects on the traffic network flow.

## Comparative results

Understanding the effects of junction removal on network connectivity is made possible by comparing the Average Connectivity Indexes (*ACIN*) and Connectivity Indexes (*CIN*) for the network junctions. The fact that $$v_{p_4}$$ and $$v_{p_2}$$ have been designated as Non-Critical Removal Nodes (*NCRNs*) in *ACIN* suggests that removing them will improve average connectivity. On average connectivity, however, $$v_{p_1}'s$$ removal has a noticeable effect, indicating its importance in the network. Furthermore, the removal of $$v_{p_3}$$ is notable for having the greatest detrimental impact on average connectivity. Upon removing $$v_{p_2}$$, there was an increase in overall connectivity, as indicated by its identification as an *NCRN* on *CIN*. Similarly, the removal of $$ v_{p_3} $$ has the greatest detrimental effect on overall and average connectivity. The disparities in connectivity metrics highlight the crucial functions of particular junctions, but it’s unclear what would happen if $$ v_{p_1}$$ or $$ v_{p_4} $$ were to disappear. Selecting the best approach relies on particular optimization objectives, but a thorough grasp of the traffic network dynamics requires taking into account the combined insights from both *ACIN* and *CIN*.

## Discussion of results

The analysis of *ACIN* and *CIN* for the network junctions reveals important insights into the impact of their removal on the overall connectivity. In terms of *ACIN*, the identification of $$v_{p_4} $$ as a *NCRN* suggests that its removal increases average connectivity, while the removal of $$ v_{p_1}$$ significantly affects the network. The highest negative impact on average connectivity is associated with the removal of $$ v_{p_3} $$. Similarly, *CIN* highlights $$ v_{p_2}$$ as an NCRN, with its removal increasing overall connectivity. The most substantial negative impact on both average and overall connectivity is attributed to the removal of $$ v_{p_3} $$. Notably, the differences in connectivity measures emphasize the critical role of specific junctions. However, the effects of removing $$ v_{p_2} $$ or $$ v_{p_4}$$ remain indeterminate. A comprehensive evaluation considering both *ACIN* and *CIN* insights is essential for making informed decisions in optimizing the traffic network.

## Advantages and limitations

As a result of our examination, the following are the main benefits and Limitations:

1. Due to the fact that neutrosophic graphs manage uncertain information with three membership graphs, the primary goal of our work is to define the idea of $CI_{N}s$ in this setting.
2. Neutrosophic graphs are described with the aid of three forms of components: membership, indeterminacy membership, and non-membership, whilst neutrosophic graphs are characterised by simplest one issue.
3. The authors have generalised the findings of $CI_{N}s$ in neutrosophic graphs in their locating. For instance, if the second feature is ignored, the outcome of $CI_{N}s$ in neutrosophic graphs appears as a particular case of their results in neutrosophic graphs.
4. In contrast, the investigation proves that neutrosophic graphs might have less facts compared with fuzzy and intutionistic fuzzy graphs.
5. Deals not only uncertainty but also indeterminacy due to unpredictable environmental disturbances.Unable to rounding up and down errors of calculations
6. Unable to handle more uncertainties.

## Conclusion

Within the neutrosophic framework, the authors of this study proposed a novel concept called Connectivity Index Numbers (*CINs*), which uses three levels of membership to address ambiguity and uncertainty. They suggested a categorization of neutrosophic graphs according to *CINs*, providing examples to help with understanding. The concept of *CIN* is extended to edge and vertex-delete neutrosophic graphs in this paper, with examples offering useful insights. The authors introduced several connection node types, such as Non-Critical Edge Removal (*NCER*), Non-Critical Removal Node (*NCRN*), and neutrosophic neural nodes, along with their corresponding results. They also defined average connectivity indices for neutrosophic graphs. The demonstration of these concepts’ applicability was conducted within the framework of a transport flow network. The study also examined real-time applications, outlining two kinds for additional research and comprehension. In general, the study makes a significant contribution to the fields of connectivity analysis and neutrosophic graphs by providing insightful new applications.

*Future Work:* In future, we will study the most general form of graph and neutrosophic graph as of today that is Super Hyper Graph and Neutrosophic Super Hyper Graph^45,46^ and to study its neutrosophic super hyper graph connectivity index and its properties. In addition to this, some fundamental results will be addressed and for better understanding examples will be provided^54–60^. We will also try to discuss

- Hyper-Neutrosophic planer graphs as well as complete neurotsophic fuzzy graphs.
- Complex super Hyper Neutrosophic graphs in decision making methods
- Super Hyper Neutrosophic graphs and its applications

## Data Availability

All the data is provided in the manuscript.
